# Modulation of Small RNA Signatures by Astrocytes on Early Neurodegeneration Stages; Implications for Biomarker Discovery

**DOI:** 10.3390/life12111720

**Published:** 2022-10-27

**Authors:** Leonardo López-Cepeda, Juan David Castro, Andrés Felipe Aristizábal-Pachón, Yeimy González-Giraldo, Andrés Pinzón, Pedro J. Puentes-Rozo, Janneth González

**Affiliations:** 1 Departamento de Nutrición y Bioquímica, Facultad de Ciencias, Pontificia Universidad Javeriana, Bogotá 110231, Colombia; 2 Laboratorio de Bioinformática y Biología de Sistemas, Universidad Nacional de Colombia, Bogotá 111321, Colombia; 3 Grupo de Neurociencias del Caribe, Unidad de Neurociencias Cognitivas, Universidad Simón Bolívar, Barranquilla 080002, Colombia; 4 Grupo de Neurociencias del Caribe, Universidad del Atlántico, Barranquilla 080007, Colombia

**Keywords:** Astrocyte Derived Extracellular Vesicles (ADEVs), small non-coding RNA (sncRNA), sncRNA transcriptome (sncRNome), circulating sncRNA, extracellular sncRNA

## Abstract

Diagnosis of neurodegenerative disease (NDD) is complex, therefore simpler, less invasive, more accurate biomarkers are needed. small non-coding RNA (sncRNA) dysregulates in NDDs and sncRNA signatures have been explored for the diagnosis of NDDs, however, the performance of previous biomarkers is still better. Astrocyte dysfunction promotes neurodegeneration and thus derived scnRNA signatures could provide a more precise way to identify of changes related to NDD course and pathogenesis, and it could be useful for the dissection of mechanistic insights operating in NDD. Often sncRNA are transported outside the cell by the action of secreted particles such as extracellular vesicles (EV), which protect sncRNA from degradation. Furthermore, EV associated sncRNA can cross the BBB to be found in easier to obtain peripheral samples, EVs also inherit cell-specific surface markers that can be used for the identification of Astrocyte Derived Extracellular Vesicles (ADEVs) in a peripheral sample. By the study of the sncRNA transported in ADEVs it is possible to identify astrocyte specific sncRNA signatures that could show astrocyte dysfunction in a more simpler manner than previous methods. However, sncRNA signatures in ADEV are not a copy of intracellular transcriptome and methodological aspects such as the yield of sncRNA produced in ADEV or the variable amount of ADEV captured after separation protocols must be considered. Here we review the role as signaling molecules of ADEV derived sncRNA dysregulated in conditions associated with risk of neurodegeneration, providing an explanation of why to choose ADEV for the identification of astrocyte-specific transcriptome. Finally, we discuss possible limitations of this approach and the need to improve the detection limits of sncRNA for the use of ADEV derived sncRNA signatures.

## 1. Introduction

Neurodegenerative diseases (NDDs) are caused by a general dysfunction of the Central Nervous System (CNS), characterized by progressive loss of neuron structure and function [1]. In recent years, NDD have become a global public health problem due in part to the increase in the elderly population in both developed and under developing countries. Pathologies such as Alzheimer’s disease (AD) (49–65%) [2,3], Parkinson’s disease (PD) (15%) [2], Vascular Dementia (VD) and Multiple Sclerosis (MS), among others, are included within the ND. Environmental factors play an essential role in the etiology of NDDs and sporadic origin is dominant; VD is an acquired disease, and sporadic cases represent more than 95% of AD, 90% of PD and 80% of MS [4,5,6]. Diagnosis of NDDs is made difficult by lack of precision and it is based in the analysis of clinical history (mainly through cognitive and motor neurological examination) [7,8], and paraclinical assessment that includes neuroimaging, and Cerebrospinal Fluid (CSF) evaluation [8,9,10]. Clinical assessment depends on symptoms, often not so apparent [7], and often it is prone to bias and lack of accuracy.

Another characteristic of NDD diagnosis is the complexity associated with paraclinical assessment which troubles timely diagnosis. Detection of distinct hallmarks of NDDs has been possible with neuroimaging and CSF evaluation up to very early time points before symptoms onset (~30 years) [11]. However, invasiveness, low availability, and risk associated with CSF measures [11], and high costs, long operating time, and inaccessibility associated with neuroimaging [8,12], limits the widespread use of these markers. Moreover, the presence of this alterations is not enough for diagnosis. Such a situation limits the success and the extent of therapies and treatments [11,13,14,15], but it is also an opportunity for simpler peripheral biomarkers which are preferred due to their lower invasiveness, risk, and complexity [16]. In support of this view successful CSF or neuroimaging biomarkers have been evaluated in peripheral fluids such as blood, saliva, plasma, serum, and tears, but markers showing promising results in CSF and neuroimaging require ultrasensitive techniques for peripheral evaluation due to reduced concentration and cross-reactivities [8]. Still with biosensor detection down to femtomolar levels [17], fluctuations non associated with disease also affect biomarker performance [18,19,20]. Recently, composite measurements of biomarkers (e.g., t-tau/Aβ42, p-tau/Aβ42, Aβ42/Aβ40) have improved the precision of peripheral based biomarkers [17], but there remains an urgency for the development of simpler cost-effective biomarkers for the detection of NDDs in early neurodegeneration stages [11,15].

Astrocytes regulate central processes for neuronal microenvironment maintenance, protection, and repair [21,22], including neuroinflammation [23], which is a hallmark in most ND. Depending on severity or duration an insult/injury can lead to astrocyte dysfunction either by impairment of the normal process or by gain of an abnormal function (astrogliosis), which could trigger neurodegeneration by neuronal microenvironment dysregulation (a process termed astrocytopathy) [24]. Astroglial pathology has been detected before symptoms onset in various NDDs [25,26,27]. Hence astrocyte dysfunction constitutes a target for early neurodegeneration biomarker research. A variety of glial-derived substances such as DJ-1, CCL2, CCL7, CXCL10, interleukins (IL-1β, IL-2, IL-6, IL-8, IL-12, IL-18), and CRP [16,28] have been studied with diagnostic interest. Interestingly some of them have shown diagnostic potential in peripheral tissues such as plasma for AD or tears for PD [16,28], and it is likely that these testing become routine screening target for NDD early diagnosis.

Another family of biomarkers deserving interest for their discriminative potential are small non-coding RNAs (sncRNAs) signatures. It is well known that micro-RNA (miRNA) dysregulation is associated with NDD and neurodegeneration [29,30]. Additionally, other sncRNAs such as piwi RNAs (piRNAs) and transfer-RNA-derived RNA (tsRNAs) have gained recent interest as potential NDD biomarkers [31,32,33]. Cells of the body actively generate and maintain a pool of circulating/extracellular sncRNA formed by contributions of Extracellular Vesicles (EV), ribonucleoprotein complexes (RNPs) and lipoprotein complexes (LPPs) [34,35], which can be studied in peripheral tissues as sncRNA signatures whose expression patterns define one or another state. Total extracellular miRNAs (RNA from all three sources), dysregulates in specific pathological contexts such as Huntington disease (HD) and VD demonstrating diagnostic value [10,36]. In NDD small RNome signatures attained VD and PD diagnosis [10,37], MS diagnostic subtyping [9], and the prodromal detection of AD [31], and HD [36], with a successful example of detection of these signatures in peripheral tissues [9]. RNA signatures constitute one of the most promising biomarkers for NDD and other diseases, which is demonstrated by their successful establishment as diagnostic and therapeutic biomarkers in breast cancer [38].

Furthermore, studying sncRNA signatures in a cell specific manner would improve the precision of peripheral signatures of sncRNA. This could be accomplished by isolating cell-specific EVs—which overpass the BBB—and studying their sncRNA content. Recently, flow cytometry separation of Astrocyte Derived Extracellular Vesicles (ADEVs) with GFAP revealed upregulation in the symptomatic phase of a MS mouse model [39]; this result exemplifies the astrocyte relevance in neuroinflammation—a process happening in AD, PD, MS, amyotrophic lateral sclerosis (ALS) and aging [40]—but it also reveals how ADEVs could be useful for NDD diagnosis when inflammatory involvement occurs. Flow cytometric GFAP and AQP4 positive EV simultaneous detection in plasma of stress-induced exhaustion disorder patients, showed upregulation of AQP4 positive ADEV, underscoring its usefulness for distinction of conditions altering astrocyte function [41] such as TBI that would precede changes related with NDD. Astrocyte function contributes to the maintenance of BBB permeability, glutamate homeostasis, and other processes dysregulated in NDD therefore, circulating astrocyte-specific sncRNA signatures could tag other pathological processes related with ND, helping in the design of biomarkers but also in the distinction from other pathological mechanisms disentangling the role of astrocytopathy (astrocyte dysfunction) in ND.

Jovicic & Gitler, (2017) [42] showed that ADEV RNA repertories differed from the found in intracellular compartments, demonstrating that exosomal extracellular transcriptome was not a simple copy of intracellular transcriptome. ADEV derived sncRNA functions as signaling effectors; this has been evidenced by expression changes in targets cells affecting viability and function [43], and selective uptake of EV by CNS cells [44]; these findings suggest that extracellular transcriptome is sensible to astrocyte dysfunction both qualitatively or quantitatively (due to the upregulation od ADEV in pathological conditions) [39]. However, to our knowledge, there are no studies yet in NDD that separate and analyze individual astrocyte-specific sncRNA signature in humans. These signatures would dysregulate in response to astrocyte functional changes as suggested by alterations in the proteomic profile of ADEV between controls and NDD [45] and in the sncRNA profile of in vitro tat stimulated astrocytes and mouse models of NDD [45,46]. Here we review the astrocyte-specific sncRNA found dysregulated in conditions associated with NDD risk and early neurodegeneration such as a proinflammatory microenvironment, traumatic brain injury (TBI), or ischemia, evidencing the effects that ADEV transported sncRNA could exert on target cells. We propose that EV-derived extracellular sncRNAs signatures could be the ultimate biomarker for early neurodegeneration stage, due to their high specificity, stability and their biological role implied in its conserved mechanism of biogenesis. In addition, we consider methodological aspects to bear in mind that would improve astrocyte-specific circulating sncRNA preparations.

## 2. Astrocyte Relevance on Early Neurodegeneration Stages

Neurodegenerative disease (ND) etiology is particularly difficult to define, for instance Aβ peptides, which are the most representative hallmark of Alzheimer’s disease (AD), have been observed to reduce oxidative stress in some circumstances, blurring the limits between pathological and physiological changes [47], therefore NDD onset is highly heterogeneous, undefined, and not well understood [48] and early phases of NDDs difficult to define. Early neurodegeneration stages can be defined in two well separate phases that vary between diseases: preclinical and prodromal [7,20,48]. Preclinical phases can be further partitioned in pre-symptomatic and early symptomatic, while the prodromal stage is characterized by symptoms not strong enough to consider a diagnosis but clearly evidenced in clinical settings [11]. Astrocyte dysfunction appears in early phases of AD as imaged in vivo by positron emission tomography (PET) of ^11^C-deuterium-L-deprenyl (^11^C-DED) and ^11^C-BU99008, both of which recognize reactive astrocytes [49,50] and have been observed in mild cognitive impairment [49], and symptomatic early PD [50]. ^11^C-DED recognize monoamine oxidase B (MAO-B) and have also been found in MS [51]. Therefore, astrocyte dysfunction biomarkers should identify early neurodegeneration stages in most NDDs before extensive neuronal damage occurs.

Astrocytes perform multifold functions some of them essential such as regulating electrochemical balance, distributing the energy uptake (meeting the high energy requirement of neurons), and accelerating detoxification which protect neurons from oxidative damage [22]. Several papers summarize astrocyte general functions and in specific contexts such as innate immunity, inflammation, and neuroprotection [21,52,53,54,55]. Astrocytes respond to CNS injury/lesion with the activation of a complex heterogenous response termed astrogliosis [24], which can lead to the activation of pivotal processes for NDD such as neuroinflammation. Since astrogliosis modifies astrocytic function (sometimes permanently), neurodegeneration can start because of both, the reduction of essential functions performed by astrocytes and an exacerbated astrocytic activity (abnormal gain of function), this is formally called astrocytophaty [24]. Growing evidence supports that astrocyte dysfunction is sufficient by itself to start secondary neurodegenerative process in early neurodegenerative stages of different NDDs.

For instance, in Alzheimer Disease (AD) abnormal astrocyte exposure to saturated fatty acids (SFF) such as stearic, linoleic, oleic and palmitic acid (PA) has been linked higher risk [56,57]. High exposures of human astrocytes to PA decreased cell viability and mitochondrial membrane potential, also producing autophagy impairment, proinflammatory cytokine overproduction (IL-1β, IL-6 and TNF-α), endoplasmic reticulum stress, and morphological changes associated with dysfunction [58,59,60]. Furthermore, neuron exposition to equivalent concentrations of PA did not generate the same effect [57,61], but when neurons were exposed to media from astrocytes induced with PA toxicity, hyperphosphorylation of tau [57], and induction of Aβ peptide production was observed [62]. Both marks associate with onset of AD and appear in early neurodegeneration stages [11,63]. 

Animal models and human also support in vitro observations situating astrocyte dysfunction as an early pathological event in AD. In animal models, High Fat Diet (HFD) robustly induced cognitive impairment in healthy rat [64] and Tg2576 mouse models of AD [65]. Tg2576 mouse also showed higher production of amyloidogenic Aβ peptides and higher γ secretase activity after HFD [65], as well as some contradictory results with improved cognitive functions and no Aβ significant burden in some studies [66]. Alterations in the fatty acid composition seem to be responsible for these contradictions [66] but sex differences could be also responsible because study with contradictory results did not account for animal sex [66]. In general, metabolic disorder models (including HFD) caused alterations in neuroinflammation and BBB function (processes key controlled by astrocytes) in both animal models (including zebra fish) and humans [67]. Lastly, high-glycemic-load diet (HGLD) exposure of healthy humans caused higher Aβ burden measured by PET and an increasing SFF concentration associated with progression to AD in human serum and brain tissue [56], therefore helping to explain relationship between dyslipidemia and related metabolic disorders with AD and recapitulating in vitro observations.

Parkinson’s Disease (PD) development is partly due to astrocyte dysfunction with evidence building up to suggest early astroglial participation in neuroinflammation and in the disruption of several neuroprotective mechanisms as partly initiator events [68]. Several of the 19 genes causative genes of mendelian forms of PD, express in equal amount in astrocytes than in neurons, with GBA, EIF4G1, VPS35, FBXO7 and PINK1, showing higher expression in astrocytes than in neurons [68,69]. Mutations of these genes in astrocytes have been shown to disrupt lipid metabolism, proliferation, glutamate uptake, cytokine regulation, neurotrophic signaling, anti-inflammatory secretion, and therefore a role in early phases of disease is possible [68]. Animal models of PD have also shown increased expression of GFAP, neuroinflammation and onset of astrogliosis before motor symptoms [68,70]. Induced by neurotoxin 6-hydroxydopamine and rotenone PD models also displayed earlier neurodegeneration and neuroprotection diminishment concomitant with astroglial dysfunction in astrocyte conditional mutation [68].

Efforts in Vascular Dementia (VD) early detection center in the identification of subclinical symptoms of cerebrovascular conditions that precede it since a stroke constitute a risk marker by itself. Most VD cases arise after vascular damage caused by smaller and less evident pathophysiological lesions such as lacunar infarct [71]. In most cases such lesions are detected by neuroimaging and therefore there remains cost and accessibility issues for their widespread use, however alternatives of detection such as neurological tests have been proposed [72]. Combined protein detection of CRP, homocistein (Hcy) and Toll-like receptor 4 (TLR4) in serum have shown biomarker value for cognitive abnormalities related with Cerebral Small Vessel Disease (SVD) which is the most common vascular abnormality associated with VD [73]. Evidence shows that these preceding vascular abnormalities induce changes in astrocyte function, including astrocyte activation, end-foot disruption, and EAAT2 and AQP4 downregulation. Hcy induced VD mouse model similarly showed astrocytic end-foot disruption concomitant to AQP4 downregulation with astrogial activation, and these astrocytic changes took place in a symptomatic phase (cognitive deficits measured in mice) after microglial activation [71]. This evidence shows that to measure astrocytic functional changes could have a biomarker value in specific stages of VD with microglial changes being more relevant for early detection, but it requires further research and other models of VD to confirm these observations.

In Multiple Sclerosis (MS), astrocyte dysfunction takes place earlier than previously believed (in glial scar formation). Evidence shows that, before lesion demyelination, astrocytes contribute to the inflammatory response that results in the recruitment of lymphocytes and regulate BBB and BSCB permeability to favor lesion [74]. Evidence in preclinical model of experimental autoimmune encephalomyelitis (EAE) strongly support astrocyte dysfunction role in early phases of MS disrupting BBB, BSCB and promoting lymphocyte recruitment. Astrocyte-specific CCL2 deletion (a central gene in BBB disruption) in mouse ameliorated MS in EAE [23,74]. Furthermore lactosylceramide (LacCer), an activator of CCL2 and powerful inflammatory molecule, is overexpressed in astrocytes of the EAE model and its inhibition suppressed neurodegeneration [74,75]. In humans, it has been found that genetic risk factors of MS cause a strong in vitro probed effect in astrocyte function that increases NF-κB signaling and chemokine liberation, which can be related with lymphocyte recruitment in prodromal stages. In addition, astrocytes perform neuroprotective roles in MS such as the recruitment of microglia in damaged myelin clearance to favor lesion repair [74] that may take place in symptomatic phases of the disease. 

Other alterations associated with increased risk of NDD cause alterations in astrocyte function. A proinflammatory microenvironment, oxygen and glucose deprivation, intracerebral hemorrhage, traumatic brain injury (TBI), Postoperative Cognitive Dysfunction (PCD) and ethanol exposure, represent conditions inducing increased risk of NDD [56,71,76,77,78,79]. Neurovascular unit astrocytes suffer atrophy of end-foot processes under ischemia, TBI, brain contusion and NDD [71,80], while morphine induces neuroinflammation and permanent cognitive deficit in a PCD rat model [77,79], in which astrocyte mediated activation of microglia possibly initiates the insult [77]. Furthermore, rat models demonstrate PD-like deficits in motor function after repeated cycles of binge-like ethanol intake [81]. Independent of the origin astrocyte dysfunction generates changes in their secretory profile including alterations in the profile of extracellular sncRNA, among which sncRNA from Astrocyte Derived Extracellular Vesicle (ADEV) is the most studied source [77,82,83,84,85,86]. Table 1 shows sncRNA from ADEV found dysregulated in conditions associated with early neurodegeneration and NDD risk in human, mouse, and rat models. These conditions include Il-1β and TNF-α stimulus [82], Hypoxic-Ischemic Brain Damage (HIBD) [87], ischemic preconditioning [84], SOD1 mutation [88], ethanol induced cell toxicity, and morphine-mediated neuroinflammatory microenvironment which is associated with PCD [77]. Therefore, astrocyte-specific circulating sncRNA could change in response to early neurodegenerative processes making early neurodegeneration associated with astrocyte dysfunction identifiable. Although studies evaluating the potential use of total extracellular ADEV derived sncRNA in the prodromal diagnosis of AD exists [37], to our knowledge no study has evaluated potential changes in expression profile of an astrocyte-specific sncRNA signal or signature.

## 3. Astrocyte Derived Extracellular sncRNA Signatures as Biomarkers for Early Neurodegeneration

Biomarkers can be classified according to their application in risk, screening, diagnostic, prognostic, monitoring and predictive/therapeutic [165,166,167], and for their widespread clinical use must be simple, cost effective and easily accessible [11]. On the contrary, current diagnostic tools for neurodegenerative disease (ND) suffer from variable reproducibility, long operating time, invasiveness, high cost, and limited accessibility [8,12] which difficult timely and accurate diagnosis. No isolated diagnostic procedure exists, with final diagnosis being made by a variable combinatorial analysis of neuropsychological testing, electrophysiological assessment, neuroimaging techniques and CSF evaluation of Aβ peptide, t-tau, p-tau, or α-synuclein, and their combinations (e.g., t-tau/Aβ42, p-tau/Aβ42, Aβ42/Aβ40) [7,8,9,10,17]. While clinical neurophysiological testing depends on the personnel expertise and has intrinsic low accuracy [7,8], invasiveness, high cost, long operating time, and limited accessibility obstacle neuroimaging and CSF evaluation implementation in preclinical and prodromal sceneries [8,12]. CSF evaluation requires lumbar puncture, a painful, invasive, complex, and expensive collecting method [168]. For that reason peripheral fluids such as blood, saliva, plasma, serum, or tears are the target of biomarker research [16], but markers that have shown promising results in neuroimaging or CSF testing [63], often show reduced efficiency when measured in peripheral tissues due to cross-reactivities and reduced concentration [8].

Misdiagnosis is also found in NDD increasing in early phases [7,11,169,170], due to comorbidity and overlapping symptoms shifting accurate diagnosis of NDD to symptomatic phases [7,11] when there is already considerable neurological damage limiting current and future therapy scope [11,13,14,15]. Therefore, there remains an urgency of biomarkers for the timely and accurate detection of NDDs in early neurodegeneration stages [11,15]. Diagnostic/screening and predictive biomarkers are also needed to aid in the development of therapies through evaluation, and when disease-modifying therapies become available for the clinical monitoring of the disease [11].

RNA signatures consist of the validation of an expression profile in a subcellular location, cell, tissue, or organism for the recognition of pathological or physiological states, and the activated cellular pathways [171]. These signatures can be exploited for biomarker purposes with great efficiency generating clinical value [172], consequently, total extracellular sncRNA role in the diagnosis of NDD has been visited by several studies [9,10,31,36,37,91,173]. CSF sample evaluation of sncRNA allowed AD and frontotemporal dementia (FTD) diagnosis as well as the prodromal identification of Huntington disease (HD) [36,153]. Furthermore, through the development of sncRNA signatures it was possible to diagnose Parkinson’s Disease (PD) (with mean disease duration = 2 years) with an AUC of 97%, [37], and mild cognitive impairment (MCI)—prodromal AD—to AD conversion with an AUC of 87% [31]. In peripheral sample, sncRNAs allowed the diagnosis and subtyping of MS in serum with an AUC efficiency >74% [9] and diagnosis of Vascular Dementia (VD) with an AUC of 94% for plasma samples [10]. Although these peripheral measures show promising AUC, the highest accuracy for NDD diagnosis with total extracellular sncRNA, was obtained by combined CSF assessment of Extracellular Vesicles (EV) derived sncRNA and pTau/Aβ42/40 ratio with an AUC of 97% [31].

Identification of cell-specific signatures would increase accuracy of sncRNA measures, by blocking the influence of confounding factors affecting circulating sncRNA expression such as changes in cell proportion and secretion [174] and can be paramount for an accurate diagnosis. Even though there are several studies using extracellular sncRNA focusing on its biomarker potential roles, no study has given insights of the astrocytic sncRNA fraction as a differential trait that allows changes associated with astrocyte dysfunction [175]. This cell-specificity would be likely to help in the disentangling of the role of astrocyte dysfunction in early stages of NDD and help in other areas of biomarker research such as prognosis, monitoring, subtyping, and disease stratification. However, this could not be possible without proper knowledge of the biology of extracellular RNA delivery mechanisms. When circulating endogenous sncRNAs were first detected in the maternal plasma of pregnant women [176] a potential application for them in the clinical field readily emerged and therefore a necessity to evaluate the stability of this sncRNA. Plasma sncRNAs was found to resist plasma RNAses, incubation at room temperature and repeated freeze thaw cycles, but synthetic RNA failure to resist degradation in human plasma allowed the identification of an RNA-protective mechanism activated in endogenous sncRNA [177]. Further research revealed at least different mechanisms associated with endogenous extracellular sncRNA resistance (carrier mechanisms); RNA ribonucleoprotein complexes (RNPs), lipoprotein complexes (LPPs) or extracellular vesicles (EV) [34].

## 4. Relevance of Carriers of sncRNA in Astrocyte Derived Extracellular sncRNA Biomarker Design for Neurodegenerative Disease (ND)

An RNA carrier is any cell derived structure known to carry extracellular RNA with a protective role of its RNA. Extracellular vesicles (EVs) participate in a safe cell-to-cell RNA delivery and according to their biogenesis can be subtyped in at least three categories: exosomes, microvesicles (MVs) and apoptotic bodies (ApoBDs) [34]. Exosome biogenesis is a complex multistep process involving endosome inward budding of membrane to form intraluminal vesicles (ILV), which accumulate to form multivesicular bodies (MVBs), MVBs fuse with plasmatic membrane causing liberation of ILV to the extracellular space as exosomes (30–150 nm) [168]. Protein complexes such as Endosomal Sorting Complex Required for Transport (ESCRT) are necessary for exosome formation and contents of exosome include cytoplasmatic proteins and sncRNA with functional capacity on target cells. Exosomes have a formal role in cell-to-cell signaling with implications for brain disease [178,179]. MV are larger EV (100–1000 nm) [168], originated by budding of plasmatic membrane after specific stimulus such as hypoxia have taken place. MV biogenesis may involve membrane curvature and destabilization, with further shedding of vesicles to the extracellular space. Alternatively, MV could also arise as a result of recruitment of exosome machinery (e.g., ESCRT) to the plasmatic membrane [180]. Astrocyte-derived MV sncRNA has been shown to target neurons increasing vulnerability to cell death by miR-34a upregulation [181]. ApoBDs are considered the most variable EVs, generally large (500–4000 nm) [168], ApoBDs are heterogenous in size and content composition carrying large quantities of RNA and, interestingly may generate their own EVs. ApoBDs membrane composition is distinct from other membrane with exposed phosphatidylserine (PS) in their outer phase and, among the unique cargo transported by ApoBDs, whole functional organelles have been found [182].

For a widespread diagnosis of neurodegenerative disease (ND), cheaper, painless, and less invasive, simpler biomarkers should be designed, especially for preclinical and prodromal sceneries. Several reports evidence that EVs originated from astrocytes (ADEV) appear in peripheral fluids such as mouse [39] or human plasma [183] and thus ADEV location in such samples is considered validated. Interestingly, transmission electron microscopy (TEM) evidenced the liberation of EVs by astrocyte end-feet. After that, these vesicles cross the endothelial cells by transcytosis to be released in small vessels both in in vitro and in a focal brain injury in vivo mouse model [184]. Thus, ADEVs which are able to cross BBB [185] represent good candidates for the development of peripheral non-invasive biomarkers.

Non vesicular carriers for sncRNA include ribonucleoprotein (RNPs) and lipoprotein (LPPs) complexes. RNPs can employ nucleophosmin or argonaute proteins, while LPPs are lipid-protein particles of varying density that subdivide into; chylomicrons (75–1200 nm), very low-density lipoproteins (VLDLs) (30–80 nm), low-density lipoproteins (LDLs) (18–25 nm), and high-density lipoproteins (HDLs) (5–12 nm) [34,186]. Extracellular RNPs carry a variety of sncRNA whose composition differs from what is found in the cell of origin [187]. HDLs and LDLs are involved in RNA regulatory signaling with differential expression of sncRNA such as miR-135a, miR-188-5p, miR-877 miR-223, miR-105, or miR-106a [34,188]. LDL and HDL capacity to cross BBB has been demonstrated [189,190], while the role of BBB in RNPs transit requires further research.

Besides having the ability to cross BBB to be found in peripheral samples, sncRNA carriers should be sortable to assign a cell-specific sncRNA signature to astrocytes. This depends on both the availability of cell markers for the cell in question and how often the carrier integrates the marker within their structure. EV formation involves budding of different membranes of the cell of origin from which they inherit cell surface markers that can be exploited [168] using antibodies in a similar fashion to FACS and with immunoprecipitation techniques. Several cell surface markers such as ACSA-1 [183], LRP1, ITGA6 [191] and EAAT-1 and 2 [192] have been reported in ADEV and need validation as sorters, while GFAP and AQP4 have been employed in high resolution flow cytometry applications to separate ADEV [39,41]. Unspecific expression of some of these markers in other cells has motivated the development of sequential separation employing two markers to increase purity of separations [41] and it is expected that validation of other cell markers increases the precision of ADEV separations, including a few astrocyte markers not evaluated yet in ADEV such as GLUL. Plasmatic membrane proteins with exposed epitopes should be preferred for preservation of EV structure and function during the process of separation. Apart from that, exosome, MV and ApoBDs possess their own specific structural biomarkers that can be exploited for EV subtyping in separation protocols [34] but such structural characteristics are shared by most cells. Unfortunately, EV yields (as measured by nanoparticle tracking analysis-NTA) often result in lowquantities of sncRNA and protein required for further characterization and sncRNA applications [187,193], and thus it is preferable to work with EV yields rather than with exosome or MVs yields. ADEV derived sncRNA however represent the ultimate candidate for the identification of astrocyte derived circulating sncRNA signal signature.

Non-vesicular carriers have more limitations regard to recognition of cell specific signatures. RNPs express ubiquitously with a 2% showing strict tissue specificity representing about 20 families of RNPs, whether isoform tissue specificity occurs and to which extent is yet to be discovered [194]. Conditions associated with RNP secretion and sncRNA association are poorly understood, as well as which ribonucleoproteins are secreted and if tissue specific RNPs are secreted. RNPs may also contaminate EVs samples but Size Exclusion Chromatography (SEC) and pretreatment with proteinase K/RNAse A have been successfully employed to exclude their presence in human sample [195]. LPPs are more abundant than EV in blood [34] and in a similar fashion to EV protein masquers in the lipid monolayer of astrocytic origin could be explored, but unfortunately biological characteristics of LPPs make them unsuitable for the identification of sncRNA signatures of astrocyte origin; Chylomicrons, VLDLs and LDLs have a peripheral origin, not an astrocyte-related origin and, similarly, HDL particles though a fraction originates in CNS this occurs by extracellularly free association of apo-AI with lipids [186]. Moreover, prominent physicochemical properties of LPPs make them contaminants in EV preparations and thus new methods for their separation are needed [34].

**Figure 1:**
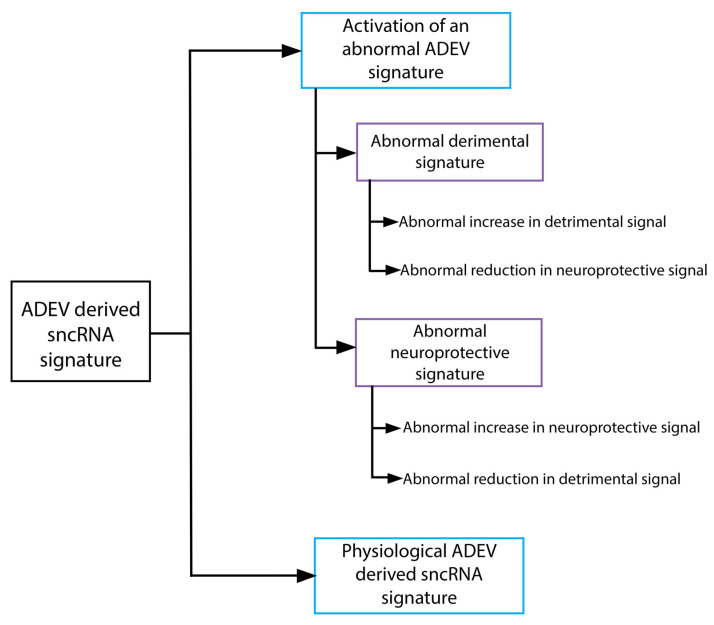
The scheme shows all expression changes that could associate with a neurodegenerative disease outcome with regard to the neuroprotective or neurodegeneration associated effects of the sncRNA involved in an ADEV-derived sncRNA signature.

The sncRNA carriers also differ in the type of sncRNA transported. While MV sncRNA resembles expression in the cell of origin, EV and RNPs sncRNA show strong differential expression [42,187]. Several sorting mechanisms for sncRNA have been proposed to explain these differences [196], thus it would be valuable to identify which sncRNA express in exosomes of astrocytes under normal conditions and pathological states. Table 1 shows ADEV derived sncRNA that dysregulated in models of CNS insult with their effect on target cells of the CNS and the periphery, according to the net effect of their upregulation on neuronal function and viability they were classified as neuroprotective or associated with increased risk of neurodegeneration (Table 2). As shown by Figure 1 several outcomes regarding neuroprotective or neurodegenerative signaling activation can be the result of neurodegenerative processes taking place in early stages of neurodegenerative disease (ND); e.g., abnormal neuroregulatory signal activation. Moreover, a neuroprotective signal can be activated and still be the result of a NDD taking place in early neurodegeneration stages. That is because underlying neurodegenerative mechanisms could be factors inducing activation of neuroprotective signaling in ADEV, and still being undetectable in ADEV because sncRNA directly related with the dysfunction are not secreted in EVs. miR-200b an ADEV derived sncRNA differentially upregulates in ADEV after ethanol induced cytotoxicity [85], being a neuroprotective suppressor of APP gene activation and Aβ production [159] (Table 1). Consequently miR-200b expression is inhibited in mouse APP Alzheimer disease models and AD patients but their upregulation in prodromal mild cognitive impairment (MCI) AD concomitant with the low expression levels in healthy controls [159] demonstrate that neuroprotective mir-200b upregulation associates with a neurodegenerative disease outcome no matter the neuroprotective status of their upregulation (Figure 1). Thus, if the expression of an ADEV derived sncRNA is differential after CNS insult, it must be considered a potential biomarker. We will explore evidence of the supposed effect of ADEV derived sncRNA dysregulated in conditions associated with risk of neurodegeneration regarding the effects on target cells and the net effect in CNS function.

## 5. Astrocyte Derived Extracellular Vesicle (ADEV) Derived sncRNA Effect on Microglia

Microglia are the resident immune cells in CNS which also contribute the most to internalize vesicles via micropinocytosis mechanisms under physiological conditions with in vitro and mouse model supporting this uptake role [44]. ADEV-derived sncRNA dysregulation often activates microglia as in morphine stimulated conditions where increased ADEV derived mir-138 liberation can stimulate the TLR7-NF-kB axis on microglia [77]. Direct activation of Toll like receptors such as TLR5, TLR7, or TLR8 are one of the most frequent effects associated with ADEV-derived sncRNA assimilation by microglia, and this can result in neuroprotective or neuromodulatory signaling [77,94,135] (Table 1). Insults to the CNS system such as Il-1β upregulate Let-7f and miR-100 [82] causing the activation of microglia by these non-canonical sequence ligand binding mechanisms, which often results in neuroprotection. On the contrary, neuroprotection caused by miR-17-5p [87] could be counterbalanced by the diminishment in Aβ clearance this sncRNA causes in microglia by targeting microglia adjacent to Aβ deposits [107,108]. Generally speaking, ADEV-derived sncRNA in microglia is related with activation of toll receptors in response to inflammatory conditions. Authors should discuss the results and how they can be interpreted from the perspective of previous studies and of the working hypotheses. The findings and their implications should be discussed in the broadest context possible. Future research directions may also be highlighted.

## 6. Astrocyte Derived Extracellular Vesicle (ADEV) Derived sncRNA Effect on Neurons

Neuronal internalization of ADEV has been reported in physiological states and multiple studies demonstrate assimilation resulting in expression changes [82,85,198]. Furthermore, after pathological stimulus, internalization of EVs by neurons can increase [199]. Neuronal death has been found to be inhibited under Hypoxic-Ischemic Brain Damage (HIBD) by miR-17-5p [87], OGD by mir-21 [116], Intermitent-hypoxia with reoxygenation (IHR) injury by miR-92b-3p [129] and Spinal Cord Injury (SCI) by miR-182 [155]. These neuroprotective sncRNA signaling is disrupted in astrocytes after Il-1β stimulation [82] and oxidative stress induced by HIBD [87].

On the other hand, the same stimulus causing the secretion of neuroprotective sncRNA on astrocyte can stimulate the secretion of miR-16-5p, which reduces neuronal function after inflammatory stimulus [82]. MiR-146 is inflammatory on neurons, and Ibáñez et al., 2019 [85], showed that neurons took this sncRNA from EV. Interestingly, this sncRNA has a short half-life in CNS (1.5–2 h) [145], which could be evidence of cell specific EV absorption.

Ischemic preconditioning elevates miR-92b-3p favoring neurite growth, viability and mitochondrial function on neuron [84]. However, conditions such as Acute Spinal Cord Injury (ASCI) or Obstructive Sleep Apnea causing IHR lesion downregulate their expression [129,130] (Table 1). It is therefore expected that astrocyte derived exosomes in these last conditions produce less miR-92b-3p generating a signal for this case of loss of a normal function.

## 7. Astrocyte Derived Extracellular Vesicle (ADEV) Derived sncRNA Effect on Other Astrocytes

Interestingly ADEV uptake by astrocytes is expected to occur in pathological conditions such as NDD [44]. Regarding this behavior neuroprotective upregulated signaling causes increase in astrocyte viability and function (Table 1 and Table 2). Astrocytes can liberate neuroprotective signaling such as miR-873a-5p after TBI which is targeted by lncRNA associated with apoptosis. On the other side it is usual that neuroprotective signals come from astrocyte subjected to damaging conditions such as ethanol-induced neuroinflammation which increases the production of miR-182 as EV astrocyte-associated cargo [85] or TBI inducing miR-873a-5p expression [86] (Table 1). The contrary is also possible, such as in the case of miR-200b which decreases its expression as vesicular cargo after ethanol, but it is neuroprotective [85].

The sncRNA effects on astrocytes can be quite dynamic. For example, miR-21 exacerbates astrocyte activation worsening optic nerve lesion [88] but causes a neuroprotective polarization of astrocytes after Ischemic spinal cord injury (ISCI) injury in neurothropic A2s astrocytes [117]. This miRNA is also produced in excess by EV of astrocytes expressing mutant SOD1 and miR-146a was shown to inhibit this overproduction [88].

Some stimuli associated with early neurodegeneration conditions such as proinflammatory cytokine secretion, lesions, and toxicity, in the “right cell” cause the secretion of context dependent neuroprotector signaling [88,146] enclosed in astrocyte-derived Extracellular Vesicles (EV). This is the case with miR-146a which in astrocytes and neural and oligodendrocyte progenitor cells (NPCs and OPCs) promotes indirectly neuronal survival and axon health. After miR-146a stimulation astrocytes restore expression of GFAP and equilibrate Ca^2+^ levels in mSOD1 mice, while in NPCs and OPCs promotes myelin production [88,146]. On the other side miR-146a proinflammatory role by stimulating TLR4 receptors and increasing innate immune response is well known in NDD such as MS, EV derived miR-146a can mediate cortical neuron inflammatory damage in ethanol-induced cytotoxicity [85,145]. Additionally, miR-146a was found to be increased by all the early neurodegeneration associated conditions studied [85,145,146] with ethanol stimulus supporting evidence of dysregulation as an EV cargo. As miR-146a is damaging for cortical neurons [85] but beneficial for astrocytes and oligodendrocytes [88,146], it is expected their effect depends on how many cells of each kind are near their origin of secretion, thus dose dynamics would be important.

## 8. Astrocyte Derived Extracellular Vesicle (ADEV) Derived sncRNA Effect on Peripheral Cells

Astrocytes are one of the most numerous glial cells in CNS and are key mediators in the neuroinflammatory process which recruits peripheral immune cells to the CNS [23], hence a systemic effect of ADEV sncRNA expression alterations is expected. Strong stimulus such as Traumatic Brain Injury (TBI), causing extreme CNS perturbations are expected to produce ADEV sncRNome alterations with an actual capacity to exert functional changes in peripheral cells with important role in CNS pathogenesis such as leucocytes. The most common example is mir-155, which is upregulated in ADEV mSOD1 mouse, and globally upregulated in Alzheimer disease (AD) [139,145], activating T-cells and dendritic cells [149].

TBI and fractures are a frequent occurrence in clinical routine (polytraumatic injuries), with TBI patients with concomitant fractures reporting and improved fracture healing process with faster and more robust regeneration, which later was supported by studies. In the finding of candidate healing molecules to improve fracture healing process various cytokines, hormones, correlated with improved healing and more importantly, serum derived of TBI patients accelerated healing by increasing cell proliferation [200]. TBI causes the downregulation of miR-16-5p [99], an ADEV-enriched sncRNA [82] negative regulator of fracture healing by reducing osteoblast differentiation and proliferation and inducing cell-cycle arrest and apoptosis [99] (Table 1).

## 9. Limitations of Small Non-Coding RNA Signatures of Astrocyte Derived Extracellular Vesicles (ADEVs) as Biomarkers of Early Neurodegeneration Cells

Tissue-specific markers such as ADEV-derived sncRNA signatures would improve signal-to-noise ratio and decrease variability of extracellular sncRNA signatures enriching for disease-specific biomarkers [34]. It would also allow the staging of NDD—because astrocyte dysfunction varies according to neurodegeneration phase—and allow the detection of early stages of NDD associated with astrocyte dysfunction. Furthermore, an astrocyte-specific signal could help in the unravel of the role of astrocyte in different NDD. However, the recovery of ADEV derived sncRNA is not free of challenges, from which the most immediate would be the low amounts of sncRNA reported in extracellular vesicles (EVs) [187]. Total EVs including microvesicles (MVs) and exosomes, but excluding large apoptotic bodies (ApoBDs), carry more than one copy of sncRNA per EV but these quantities are far from being numerous; miRNAs are present in one copy per EV, and specific miRNA such as miR-21 in about 1 copy per 10 EVs. The most abundant class of sncRNA found in EVs were small nuclear RNAs (snRNAs) such as U2 snRNA [187], which although with some relevance in neurodegeneration [201] have a role not comparable to other sncRNA classes such as miRNA. A highly selective sncRNA sorting machinery have to exist for these EV derived miRNAs to exert a function in target cells [187], and fortunately some protein candidates involved in this process have been found [196]. Moreover, not so large quantities of miR-21 are required to reduce the expression of antagonists in cell targets as seen in more astringent in vitro models [187] which underscores the need to develop more sensitive methods for the detection of such sncRNA with functional effects in cell targets that would be relevant for biomarker and treatment purposes.

**Figure 2:**
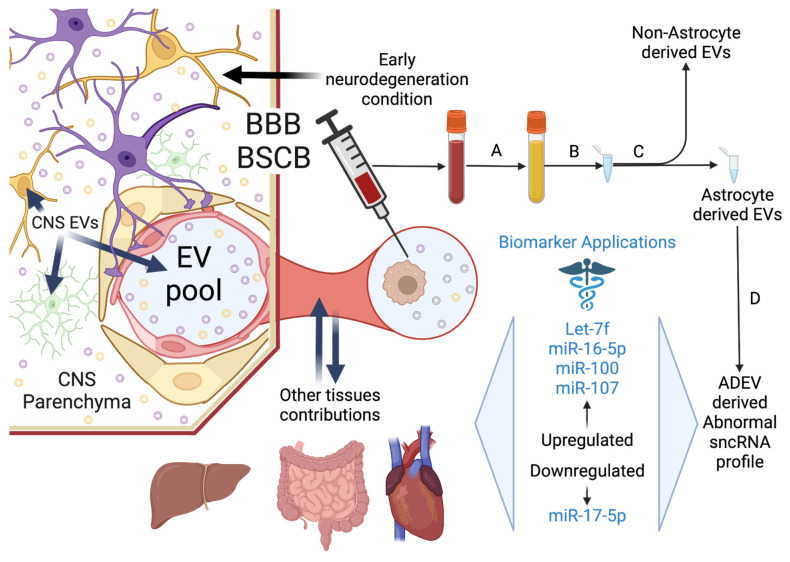
The astrocyte-derived extracellular vesicle (ADEV) derived sncRNA journey; from secretion to sncRNA isolation and analysis. Astrocytes secrete vesicles which composition changes in response to stimulus (e.g., TNF-α signaling due to chronic insult). In CNS ADEV integrate an extracellular pool of total CNS-EV made up of neuron-derived extracellular vesicles (NDEV), microglia derived (MDEV) and oligodendrocyte derived (ODEV) among others. A fraction of ADEVs is taken up by microglia and neurons, but some of them scape across the BBB or BSCB depending on the location, arriving to peripheral tissue, where again a fraction may be internalized by peripheral cells that also contribute with their own EVs increasing signal noise and reducing signal intensity. Leukocytes possibly internalize ADEVs, but the rate of this process probably changes along the disease state. (**A**) ADEVs represent a small fraction of EVs in the peripheral pool (e.g., NDEV constitute about 1% of total blood EVs), and they must be taken up in the biofluid to be sampled, generally plasma. (**B**) Enrichment concentrate EV and particles with similar physicochemical properties in a small volume allowing ADEV to accumulate from regular sample volumes (**C**), separation concentrates ADEV and their specific sncRNA signal in a sample and this implies using antibodies to exploit astrocyte specific surface markers. (**D**) Finally, sncRNA must be isolated to identify a cell-specific signal. The drawing shows a hypothetic dysregulation signature representing NDD in an early neurodegeneration phase with abnormal activation of neuroprotective signaling.

Although bodily fluids are expected to have a higher amount of EV this is not always the case. EVs counts as measured by nanoparticle tracking analysis (NTA) reveals yields of 10^6^ particles/mL for human CSF [173], 10^10^ particles/mL for mouse plasma [202], and 6 × 10^9^ particles/mL for mesenchymal stem cell derived EVs in culture [203]. Variability in EV separation protocols is expected to influence these results underscoring the need to develop higher yield capabilities in specific methods for EV separation, bearing in mind that internalization will reduce the EVs produced by CNS cells including astrocyte-derived EVs (ADEVs) collected in peripheral samples (Figure 2). Internalization of EV uptake occurs in CNS carried out mainly by microglia followed by neurons [44] but, considering the functions that some ADEV produced sncRNA such as miR-155 carry on immunological targets outside CNS such as leucocytes (Table 1) [151], which have a great capacity to internalize vesicles through phagocytosis, it is possible that another fraction of ADEV is lost by peripheral tissues internalization in healthy or diseased states (Figure 2). EV production seems to increase under stress conditions [204] and it is possible that upregulated EV production increases the sncRNA copy number in a dose-dependent manner generating a detectable and measurable signal that could be approached by widespread techniques such as qPCR carried out in ADEV derived sncRNA samples.

In addition, current separation protocols seem to fail in the recovery of the total EV load produced by the cell, with variable results depending on the method representing no consensus in a gold standard separation method [193,203]. Methods for EV isolation can produce a high yield but low specificity such as polymer precipitation-based technique (Peg) and ultracentrifugation (UF) or a high specificity and low yield which include coupled methodologies that may include filtration coupled to size exclusion chromatography (SEC) or immunoprecipitation coupled to UF. Therefore, a non-specific fraction is lost in coupled methodologies that do not fully achieve the separation of non-vesicular entities due to shared physicochemical characteristics [34,193] and this includes particles such as lipoproteins (LPPs) that carry sncRNA to interfere with sncRNA signal. Cell-specific vesicular isolation of astrocytes is possible by employing cell-specific surface markers [39] that obligatorily depend on antibodies against these markers. It should be noted that EV-derived sncRNA is theoretically the only source of sncRNA whose cell or tissue of origin can be identified due to these cell markers, but some consideration should be taken. First the epitopes of these antibodies should localize in the external face of the vesicle and thus membrane markers should be preferred to avoid artifactual separations with immunoprecipitation techniques, secondly the total isolation of ADEV derived sncRNA signature is not possible but deconvolution techniques have been used before to separate sncRNA signals from different cellular origins and should work considering that the signal is more specific that multiple cellular signals.

## 10. Conclusions

Biomarkers for neurodegenerative disease are a necessity but their design is challenging. We briefly reviewed the central role of astrocyte dysfunction in the course and development of neurodegenerative disease (NDD) and how sncRNA changes in response to astrocyte function could regulate function on target cells. Different biomarker applications such as the diagnosis in early stages of neurodegeneration would be possible by employing astrocyte specific signatures. Such developments could take part in future molecular diagnostics whose development is expected to improve treatments supporting true neuroprotective/neurorestorative therapies through early diagnosis.

However, several methodological aspects should be considered to make ADEV-derived sncRNA a reliable biomarker. New ADEV separation protocols have to increase EV yields without increase contamination with both other particles such as lipoprotein complexes or non-astrocytic EVs. Furthermore, if contamination remains deconvolution techniques may be explored to diminish noisy signal of circulating RNA in complex with RNPs and lipoprotein. Small nuclear (snRNA) is the most abundant class of sncRNA in EVs samples but this should be confirmed in astrocytes and small quantities of miRNA have been shown to influence target cell function and we should employ sensitive enough techniques to detect these small increases. Furthermore, pathological conditions and stress tend to increase EV production and it could generate a dose dependent signal of sncRNA that responds to an early neurodegeneration condition.

Astrocyte-derived extracellular RNA binding protein (RBP) role in the functional delivery of sncRNA should be explored in near future. A total of 2% of RBP genes in human genome with cell expression specificity represent enough specificity to develop astrocyte specific detection against epitopes expressed in astrocyte expressed RNPs due to the large quantity of RBP genes. Further studies in RBP biology and function in relation with secretion are necessary to determine basic question such as whether secretion of tissue exclusive RNPs occurs. The same techniques employed for the isolation of EV should be useful for the enrichment of astrocyte derived RNPs due to their shared physicochemical properties. Because of these similarities it should be easier for research groups working in vesicular area shifting to explore RNPs role in sncRNA delivery.

sncRNA has a functional role as extracellular vesicle cargo that we are beginning to understand, thus we must improve sncRNA detection capabilities considering the relatively lows amount of ADEV derived sncRNA reported by different studies in human body fluids and the EV lost in methodological procedures before a cell specific signal can be generated.

## Figures and Tables

**Table 1:** Astrocyte Derived Extracellular Vesicle (ADEV) derived sncRNA dysregulated in conditions associated with increased risk of neurodegenerative disease (NDD) and their possible effects on cell targets based on evidence in human, as well on rat and mouse models. The third column specifies the change in expression favored by conditions associated with increased risk of ND. ALS: Amyotrophic lateral sclerosis, ASCI: Acute Spinal Cord Injury, CRC: colorectal cancer, EAE: Experimental Autoimmune Encephalomyelitis, HAND: HIV associated neurocognitive disorders, HFD: High-fat diet, HIBD: Hypoxic-Ischemic Brain Damage, IBZ: Ischemic Boundary Zone, NPC: Neural Progenitor Cells, OSA: Obstructive Sleep Apnea, PASMCs: Pulmonary Artery Smooth Muscle Cells, pMCAO: permanent Mid Cerebral Artery Occlusion, SMA: Spinal Muscular Atrophy, SCI: Spinal Cord Injury, TBI: Traumatic brain Injury.

sncRNA	Condition	Role Associated with Early Neurodegeneration Conditions or Risk of Neurodegeneration	Role in Neurodegenerative Disease or Effects in CNS Cells	Additional Roles and Effects in Peripheral Cells
Let-7f	Il-1β stimulation, ischemia	Upregulated in ADEV after Il-1β stimulation in primary rat astrocytes [82], upregulated in rat pMCAO ischemia model after lesion [89]	Sporadic ALS downregulated biomarker [90], upregulated in AD hippocampus [91]. Targets involved in FoxO and MAPK signaling pathways and apoptosis [90]. Increased after HFD in rat [92]. • Promotes differentiation in rat neural stem cells [93]. Downregulates NDRG3 expression in rat cortical neurons to regulate hypoxia response (proapoptotic) [89]. ‡ Activate TLR7 [94]. ⁑ Downregulated in glioma cell lines, inhibits proliferation and migration, increases apoptosis [95]. ∞, * Protection against oxidative damage [96]. ∞, ‡	Tumor suppressor miRNA, targets the aromatase gene (CYP19A1) [97]. ∞
miR-16-5p	SCI, TBI, Il-1β and TNF-α stimulation	Upregulated in ADEV after Il-1β and TNF-α stimulation in primary rat astrocytes [82]. Upregulated in rat tissue after SCI [98]. Downregulated in human serum and mouse model after TBI [99].	Biomarker, dysregulated in ALS serum, showed lower expression in slower progressing ALS [100]. Key implication in subacute stage of SCI [101]. • Reduce dendritic complexity and growth, spike rates and burst activity after inflammatory stimulus. Downregulates NTRK3 and Bcl2 [82]. Stimulates apoptosis and inflammatory proteins, Targets Apelin-13 inactivating ERK1/2 pathway [98]. ‡ Increases apoptosis, causes inflammation [102]. ⁑ Downregulated in glioma cell lines, targets TLN1 to increase glioma viability, proliferation, migration and invasion after TIIA [103]. ∞, *	Decreases fracture healing. Negatively regulates Bcl-2 and Cyclin-D1, therefore suppressing osteogenic differentiation, and osteoblast proliferation and survival. Inhibited proliferation promoting cell-cycle arrest and apoptosis [99]. π
miR-17-5p	OGD, HIBD	Downregulated in ADEV after HIBD in rat [87].	Potential regulator of robust differentially expressed genes causing downregulation of GABAergic synapse and signaling pathways in AD [104]. It also counter IRE1a pathway downregulating TXNIP, NL3P inflammasome activation and Il-1β production [105]. Reduces inflammation related proteins after HIBD with lower production of TNF-α and IL-1β [87]. • Reduces neuronal death and apoptosis after HIBD [87]. ‡ Promotes proliferation of activated astrocytes after SCI [106]. * Upregulated in AD, in microglia adjacent to Aβ deposits. Targets autophagy receptor NBR1 inhibiting clearance of Aβ [107,108]. ⁑ Targets APP expression [109], BNIP2, SOD, GSH-Px and CAT expression, and reduces apoptosis and inflammation after OGD [87]. ∞, ⁑	Expression increased after myocardial infarction. Inhibition associated with cardiomyocyte survival through STAT3 targeting [110]. γ Promotes osteoclastogenesis via targeting PTEN [111]. Promotes osteogenic differentiation and ossification, and cytokines such as VEGF [112]. π Upregulated in various cancers, Reduced proliferation in GIST tissue samples, targets KIT expression [113]. ∞
mir-21	Ischemia	Identified in ADEV from ALS mSOD1 mouse model with neurodegeneration stage not specified, upregulated [88]. Overexpressed in hippocampus after ischemia in rat [114].	Reduced expression in axons in alcoholism and depression [115]. Suppresses OGD induced apoptosis, and Faslg pro-apoptotic factor levels. Upregulated in neurons of the IBZ [116]. ‡ Upregulation in mSOD1 ADEV mouse model is stopped after miR-146a induction [88]. After ISCI injury, stimulates polarization of reactive neurotrophic neuroprotective astrocytes [117]. * Repress FasL in microglia o inhibit neurotoxic hypoxia activated microglia [118]. ⁑ Diminish apoptosis modulating tumor suppressor PDC4I3K/AKT/GSK-3β, including apoptosis triggered by neurotoxic Aβ_1–42_ in SH-SY5Y [119]. PTEN independent oncogene [115]. ∞ ‡	Upregulates VEGF promoting angiogenesis in transformed and non-transformed non CNS tumoral cell lines [120]. ∞
miR-30b-5p	Ischemia	Upregulated in ADEV under IPC in rat [84].	Differentially upregulated in MS, associated with non-progressive forms of the disease [9]. Downregulated in ALS [121]. Elevated in PD 6- hydroxydopamine induced rat models [122]. •	Involved in diabetic retinopathy with possible biomarker applications, regulates angiogenesis [123]. ⸰ Targets SIRT1 inhibiting autophagy of the mitochondria [122]. Suppresses lysosomal biogenesis and autophagy by inhibiting TFEB targets pre-transcriptionally [124]. ∞
miR-32	HIBD	Downregulated in ADEV after HIBD in rat [87].	Involved in the maintenance of myelin fine tuning SLC45A3 and CLDN-11 expression [125]. ˟ Downregulated in glioma, targeted ABCC4 and EZH2, it reduces proliferation and migration [126]. ∞, *	Upregulated in some cancer tissues, reduces apoptosis and promotes proliferation and migration targeting OTUD3 and promoting MYC [127,128]. ∞
miR-92b-3p	Ischemia, OSA, ASCI	Upregulated in ADEV under IPC in rat [84].	Reduces inflammation after ischemic stroke. Downregulated in OSA, apnea, hypopnea [129]. Downregulated after ASCI, promotes functional recovery after ASCI [130]. • Diminishes apoptosis, cell death, mitochondrial dysfunction and favors neurite growth [84,130], including IHR induced apoptosis. Decreases ROS production, MAOA hyperactivity and PTEN expression. Promotes phosphorylation of AKT, and GAP43 and NF-200 expression [129,130]. ‡ Inhibits IHR-induced NF-κB1, PTGS1, TNF-α, and TGF-β expression [129]. +	Downregulated under hypoxia conditions in PASMCs, reverse proliferation and cell cycle induced under hypoxia conditions [131]. ζ Inhibits IHR-induced apoptosis and CXCL5 and ADRB1 expression [129]. ∞, α Regulate proliferation, apoptosis, differentiation, and metastasis [131]. ∞
mir-100	Il-1β stimulation	Upregulated in ADEV under Il-1β and ATP induction in primary rat astrocytes [82].	Downregulated in autosomal recessive NDD SMA [132,133]. ⸸ Induces apoptosis in retinal ganglion cells exposed to H_2_O_2_ [134]. ‡ Activates TLR8 receptors post-translationally causing indirect neuronal microenvironment dysregulation, activates cytokine and chemokine release [135]. Downregulates in activated microglia, ameliorates motor function loss after SCI by targeting TLR4 and NF-κB [136]. ⁑	Activates cytokine and chemokine release in macrophages [135]. α Downregulated in hypoxia induced proliferation of PASMCs, suppress mTOR expression leading to inhibition of proliferation [137]. ζ Overexpressed in EVs from CRC cells mutant KRAS expressing [138]. ∞, ο
miR-107	TNF-α stimulation	Upregulated in ADEV after TNF-α stimulation in primary rat astrocytes [82].	Involved in AD, targets BACE1, CDK5, ADAM1, increases neuronal differentiation [139]. • Higher expression correlates with lower overall patient survival in high grade gliomas [140]. ∞	Alter key aggressiveness characteristics of prostate cancer cells such as proliferation, modulates lipid metabolism, adjacent non-tumoral tissue shows downregulation. Its expression in cancer correlates with levels in plasma [141]. ∞
mir-138	Morphine-mediated neuroinflammatory microenvironment	Upregulated in ADEV under morphine stimulated conditions in mouse [77].	Activates astrocytes induced by Tat in HAND [142]. * Internalizes mir-138 charged ADEV. Activation of microglia through direct activation of the TLR7-NF-kB axis [77]. ⁑ Promotes early differentiation of oligodendrocytes [143]. ˟	Inhibits adipocyte differentiation reducing EZH2 expression [144]. μ
miR-146a	Early asymptomatic mSOD1 ALS model, ethanol activated neuroinflammation	Upregulated in ADEV after ethanol induction in mouse astrocytes [85], Identified in ADEV from ALS mSOD1 mouse model with neurodegeneration stage not specified, downregulated [88].	Involvement in pathogenesis of MS, AD, prion disease, neurotropic virus and metal sulfate induced toxicity [145] and neuroprotective in specific contexts in ALS and stroke [88,146]. • Upregulated after stroke in NPC. Increases myelinization protein expression and differentiation towards oligodendrocyte lineage [146] ⸸, ˟ Increases inflammation and its own expression in cortical neurons [85]. ‡ Attenuates miR-21 and miR-155 expression in ALS mSOD1 mouse model, decreases astrocyte reactivity and decreases proinflammatory miRNA associated exosomal cargo production [88]. Downregulated in ALS [147]. *	Downregulated by infection (RCV virus), it diminish TRAF-6 expression, JNK activation and lung inflammatory infiltration, reduces L-1β, IL -6 and TNF-α production [148]. ε
miR-155	EAE Neuroinflammation, proinflammatory cytokines	Identified in ADEV from ALS mSOD1 mouse model with neurodegeneration stage not specified, upregulated [88]. Downregulated in spinal cord of mice with EAE [149].	Globally upregulated in AD, related with inflammation targets CFH [139,145]. Upregulated in EAE MS mouse model [150], it expression is very high in MS lesions, favors proinflammatory conditions and negatively regulates BBB [145,149]. • Downregulated in ADEV in ALS mSOD1 mouse model by miR-146a induction [88] * Upregulated by proinflammatory cytokines [88]. Downregulates fatty acid metabolism associated genes [149]. +	Increases macrophage migration, mediates activation of mononuclear phagocytes, [151]. α Constitutively highly expressed [145], promotes differentiation of TH17 cells and activation of T-cells and dendritic cells [149]. δ ROS diminish miR-155-5p expression in tumor exosomes leading to immunosuppressive tumor growth [151]. ∞
mir-182	SCI, HIBD, LPS Ethanol induced neuro-inflammation	Downregulated in ADEV after HIBD in rat [87], upregulated in ADEV after ethanol induction in mouse astrocytes [85]. Downregulated in SCI of mice [152].	Anti-inflammatory miR [152]. After ischaemia exacerbates BBB dysfunction [153]. • Enriched in neurons. Increased dendrite tree complexity, axon and neurite outgrowth, favoring expression of neurofilament-M and neurofilament-L, and AKT phosphorylation [154]. Improves SCI reducing apoptosis [152]. ⸸, ‡ Increases in ethanol-treated wild type astrocytes in a TLR4-dependent response [85]. * Inflammatory suppressor (downregulates TNF-α, IL-6, IL-1β), apoptosis reduction by caspase-3 downregulation. Decreases expression after LPS [152]. ∞, ⁑ Targeted MTSS1 tumor suppressor transcript to inhibit proliferation and migration in glioma [155]. ∞	Inhibited apoptosis regulating PDCD4 and PACS2, under non-ischemic heart failure [156]. γ Downregulated after Ischemia Reperfusion (I/R). It reduces autophagy stimulating mTOR and targeting Deptor, thus reducing lesion area after I/R [157]. ο Biomarker in prostate cancer [158]. ∞
mir-200b	Ethanol induced neuro-inflammation	Upregulated in ADEV after ethanol induction in mouse astrocytes [85], downregulated in ADEV after HIBD in rat [87].	Targets APP gene downregulating amyloid beta (Aβ), however Aβ42 inhibits its expression (possibly halts AD progression) [159]. • Increases in ethanol treated wild type astrocytes in a TLR4-dependent response [85]. * Upregulated by Aβ, its transfection reduces Aβ secretion in conditioned media, relieve memory impairments and downregulates targets such as IRS-1pSer potentially diminishing insulin resistance [160]. ‡	Under glucotoxicity increases apoptosis of human retinal pigment epithelial cells [161]. ε
miR-873a-5p	TBI	Upregulated after TBI induction in mouse [86].	Improve neurological deficits associated with TBI, exhibiting a neuroprotective role regulating inflammatory signals [86]. Targets A20 (TNFAIP3) and targeted by HOTAIRM1 a miR-sponge associated with neuronal apoptosis [162,163]. • Released by activated astrocytes [86]. * Decreases ERK and NF-κB p65 phosphorylation inhibiting LPS induced M1 phenotype and inflammatory signaling, promotes microglia M2 polarization after TBI [86]. ⁑	Proapoptotic inhibitor of cell growth, downregulated in glioblastoma [164]. ∞

Target cells: ‡—Neurons, *—Astrocytes, ⁑—Microglia, ˟—Oligodendrocyte/OPCs, ⸸—Neural Progenitor/Neural Stem cell, +—Endotelial cells, •—CNS non-identified tissue, α—Monocyte/Macrophage, γ—Cardiomyocytes, δ—Limphocytes, ε—Epithelial cells, ζ—Smooth Muscle cells, μ—Preadipocyte/Adipocyte, ο—Enterocytes/Intestinal mucosa epitelial, π—Osteoblasts/Osteogenic precursor, ∞—Cancer, ⸰—non-specified peripheral origin.3. Astrocyte Derived Extracellular sncRNA Signatures as Biomarkers for Early Neurodegeneration.

**Table 2:** Astrocyte Derived Extracellular Vesicle (ADEV) derived sncRNA dysregulated in conditions associated with increased risk of ND, categorized as neuroprotective or neurodegenerative according to the effects of their upregulation in CNS function (fourth column in Table 1) as evaluated by functional studies in model organism, human or in vitro evidence of cell function impairment of CNS cells. sncRNA differential expression could activate mechanisms of pathogenesis such as BBB and BSCB leakage and autophagy impairment or neuroprotective mechanisms such as the regulation of inflammatory signaling depending on the sncRNA.

sncRNA	Neuroprotection	Neurodegeneration
Let-7f	[93,96]	
miR-16-5p		[102,197]
miR-17-5p	[87,105]	[108]
mir-21	[116,117,119]	
miR-30b-5p		[122]
miR-32	[125]	
miR-92b-3p	[84,129,130]	
mir-100	[136]	[134,135]
miR-107	[139]	
mir-138		[76,142]
miR-146a	[88,146]	[85,145]
miR-155		[88,145,149,151]
mir-182	[152,154]	[153]
mir-200b	[159,160]	
miR-873a-5p	[86]	

## Data Availability

Not applicable.

## References

[1] Catanesi M., D’Angelo M., Tupone M.G., Benedetti E., Giordano A., Castelli V., Cimini A. (2020). MicroRNAs Dysregulation and Mitochondrial Dysfunction in Neurodegenerative Diseases. Int. J. Mol. Sci..

[2] Feigin V.L., Nichols E., Alam T., Bannick M.S., Beghi E., Blake N., Culpepper W.J., Dorsey E.R., Elbaz A., Ellenbogen R.G. (2019). Global, Regional, and National Burden of Neurological Disorders, 1990–2016: A Systematic Analysis for the Global Burden of Disease Study 2016. Lancet Neurol..

[3] Erkkinen M.G., Kim M.O., Geschwind M.D. (2018). Clinical Neurology and Epidemiology of the Major Neurodegenerative Diseases. Cold Spring Harb. Perspect. Biol..

[4] Sandor C., Honti F., Haerty W., Szewczyk-Krolikowski K., Tomlinson P., Evetts S., Millin S., Keane T., McCarthy S.A., Durbin R. (2017). Whole-Exome Sequencing of 228 Patients with Sporadic Parkinson’s Disease. Sci. Rep..

[5] Syeda T., Cannon J.R. (2021). Environmental Exposures and the Etiopathogenesis of Alzheimer’s Disease: The Potential Role of BACE1 as a Critical Neurotoxic Target. J. Biochem. Mol. Toxicol..

[6] Steenhof M., Stenager E., Nielsen N.M., Kyvik K., Möller S., Hertz J.M. (2019). Familial Multiple Sclerosis Patients Have a Shorter Delay in Diagnosis than Sporadic Cases. Mult. Scler. Relat. Disord..

[7] Bloem B.R., Okun M.S., Klein C. (2021). Parkinson’s Disease. Lancet.

[8] Hao N., Wang Z., Liu P., Becker R., Yang S., Yang K., Pei Z., Zhang P., Xia J., Shen L. (2022). Acoustofluidic Multimodal Diagnostic System for Alzheimer’s Disease. Biosens. Bioelectron..

[9] Ebrahimkhani S., Vafaee F., Young P.E., Hur S.S.J., Hawke S., Devenney E., Beadnall H., Barnett M.H., Suter C.M., Buckland M.E. (2017). Exosomal MicroRNA Signatures in Multiple Sclerosis Reflect Disease Status. Sci. Rep..

[10] Prabhakar P., Retnaswami S., Christopher R. (2017). Circulating MicroRNAs as Potential Biomarkers for the Identi Fi Cation of Vascular Dementia Due to Cerebral Small Vessel Disease. Age Ageing.

[11] Hansson O. (2021). Biomarkers for Neurodegenerative Diseases. Nat. Med..

[12] Brazaca L.C., Sampaio I., Zucolotto V., Janegitz B.C. (2020). Applications of Biosensors in Alzheimer’s Disease Diagnosis. Talanta.

[13] Swarbrick S., Wragg N., Ghosh S., Stolzing A. (2019). Systematic Review of MiRNA as Biomarkers in Alzheimer’s Disease. Mol. Neurobiol..

[14] Katsuno M., Sahashi K., Iguchi Y., Hashizume A. (2018). Preclinical Progression of Neurodegenerative Diseases. Nagoya J. Med. Sci..

[15] Le W., Dong J., Li S., Korczyn A.D. (2017). Can Biomarkers Help the Early Diagnosis of Parkinson’s Disease?. Neurosci. Bull..

[16] Nandi S.K., Singh D., Upadhay J., Gupta N., Dhiman N., Mittal S.K., Mahindroo N. (2021). Identification of Tear-Based Protein and Non-Protein Biomarkers: Its Application in Diagnosis of Human Diseases Using Biosensors. Int. J. Biol. Macromol..

[17] Kim K., Kim M.J., Kim D.W., Kim S.Y., Park S., Park C.B. (2020). Clinically Accurate Diagnosis of Alzheimer’s Disease via Multiplexed Sensing of Core Biomarkers in Human Plasma. Nat. Commun..

[18] Tan R., Wang Y., Mi X., Li H., Tu Y. (2022). A Dual-Screening Electrochemiluminescent Aptasensor Based on a Mesoporous Silica Nano-Sieve for Specific Detection of Amyloid-β Monomer. Sens. Actuators B Chem..

[19] Bateman R.J., Wen G., Morris J.C., Holtzman D.M. (2007). Fluctuations of CSF Amyloid-β Levels. Neurology.

[20] Knopman D.S., Amieva H., Petersen R.C., Chételat G., Holtzman D.M., Hyman B.T., Nixon R.A., Jones D.T. (2021). Alzheimer Disease. Nat. Rev. Dis. Prim..

[21] Souza D.G., Almeida R.F., Souza D.O., Zimmer E.R. (2019). The Astrocyte Biochemistry. Semin. Cell Dev. Biol..

[22] Colangelo A.M., Alberghina L., Papa M. (2014). Astrogliosis as a Therapeutic Target for Neurodegenerative Diseases. Neurosci. Lett..

[23] Mozafari N., Ashrafi H., Azadi A. (2021). Targeted Drug Delivery Systems to Control Neuroinflammation in Central Nervous System Disorders. J. Drug Deliv. Sci. Technol..

[24] Sofroniew M.V. (2015). Astrogliosis. Cold Spring Harb. Perspect. Biol..

[25] Kuter K., Olech Ł., Głowacka U., Paleczna M. (2019). Astrocyte Support Is Important for the Compensatory Potential of the Nigrostriatal System Neurons during Early Neurodegeneration. J. Neurochem..

[26] Guo T., Zhang D., Zeng Y., Huang T.Y., Xu H., Zhao Y. (2020). Molecular and Cellular Mechanisms Underlying the Pathogenesis of Alzheimer’s Disease. Mol. Neurodegener..

[27] Maragakis N.J., Rothstein J.D. (2006). Mechanisms of Disease: Astrocytes in Neurodegenerative Disease. Nat. Clin. Pract. Neurol..

[28] Su C., Zhao K., Xia H., Xu Y. (2019). Peripheral Inflammatory Biomarkers in Alzheimer’s Disease and Mild Cognitive Impairment: A Systematic Review and Meta-Analysis. Psychogeriatrics.

[29] Juźwik C.A., Drake S.S., Zhang Y., Paradis-Isler N., Sylvester A., Amar-Zifkin A., Douglas C., Morquette B., Moore C.S., Fournier A.E. (2019). MicroRNA Dysregulation in Neurodegenerative Diseases: A Systematic Review. Prog. Neurobiol..

[30] Gasecka A., Siwik D., Gajewska M., Jaguszewski M.J., Mazurek T., Filipiak K.J., Postuła M., Eyileten C. (2020). Early Biomarkers of Neurodegenerative and Neurovascular Disorders in Diabetes. J. Clin. Med..

[31] Jain G., Stuendl A., Rao P., Berulava T., Pena Centeno T., Kaurani L., Burkhardt S., Delalle I., Kornhuber J., Hüll M. (2019). A Combined MiRNA–PiRNA Signature to Detect Alzheimer’s Disease. Transl. Psychiatry.

[32] Qin C., Xu P.P., Zhang X., Zhang C., Liu C.B., Yang D.G., Gao F., Yang M.L., Du L.J., Li J.J. (2020). Pathological Significance of TRNA-Derived Small RNAs in Neurological Disorders. Neural Regen. Res..

[33] Prehn J.H.M., Jirström E. (2020). Angiogenin and TRNA Fragments in Parkinson’s Disease and Neurodegeneration. Acta Pharmacol. Sin..

[34] Das S., Abdel-Mageed A.B., Adamidi C., Adelson P.D., Akat K.M., Alsop E., Ansel K.M., Arango J., Aronin N., Avsaroglu S.K. (2019). The Extracellular RNA Communication Consortium: Establishing Foundational Knowledge and Technologies for Extracellular RNA Research. Cell.

[35] Szilágyi M., Pös O., Márton É., Buglyó G., Soltész B., Keserű J., Penyige A., Szemes T., Nagy B. (2020). Circulating Cell-Free Nucleic Acids: Main Characteristics and Clinical Application. Int. J. Mol. Sci..

[36] Reed E.R., Latourelle J.C., Bockholt J.H., Bregu J., Smock J., Paulsen J.S., Myers R.H. (2018). MicroRNAs in CSF as Prodromal Biomarkers for Huntington Disease in the PREDICT-HD Study. Neurology.

[37] dos Santos C.M.T., Barreto-Sanz M.A., Coreira B.R.S., Bell R., Widnall C., Perez L.T., Berteau C., Schulte C., Scheller D., Berg D. (2018). MiRNA-Based Signatures in Cerebrospinal Fluid as Potential Diagnostic Tools for Early Stage Parkinson’s Disease. Oncotarget.

[38] Griguolo G., Bottosso M., Vernaci G., Miglietta F., Dieci M.V., Guarneri V. (2022). Gene-Expression Signatures to Inform Neoadjuvant Treatment Decision in HR+/HER2− Breast Cancer: Available Evidence and Clinical Implications. Cancer Treat. Rev..

[39] Willis C.M., Ménoret A., Jellison E.R., Nicaise A.M., Vella A.T., Crocker S.J. (2017). A Refined Bead-Free Method to Identify Astrocytic Exosomes in Primary Glial Cultures and Blood Plasma. Front. Neurosci..

[40] Mayne K., White J.A., McMurran C.E., Rivera F.J., de la Fuente A.G. (2020). Aging and Neurodegenerative Disease: Is the Adaptive Immune System a Friend or Foe?. Front. Aging Neurosci..

[41] Wallensten J., Nager A., Åsberg M., Borg K., Beser A., Wilczek A., Mobarrez F. (2021). Leakage of Astrocyte-Derived Extracellular Vesicles in Stress-Induced Exhaustion Disorder: A Cross-Sectional Study. Sci. Rep..

[42] Jovicic A., Gitler A.D. (2017). Distinct Repertoires of MicroRNAs Present in Mouse Astrocytes Compared to Astrocytesecreted Exosomes. PLoS ONE.

[43] Lafourcade C., Ramírez J.P., Luarte A., Fernández A., Wyneken U. (2016). MIRNAS in Astrocyte-Derived Exosomes as Possible Mediators of Neuronal Plasticity: Supplementary Issue: Brain Plasticity and Repair. J. Exp. Neurosci..

[44] Ogaki A., Ikegaya Y., Koyama R. (2021). Extracellular Vesicles Taken up by Astrocytes. Int. J. Mol. Sci..

[45] Upadhya R., Zingg W., Shetty S., Shetty A.K. (2020). Astrocyte-Derived Extracellular Vesicles: Neuroreparative Properties and Role in the Pathogenesis of Neurodegenerative Disorders. J. Control. Release.

[46] Yang L., Niu F., Yao H., Liao K., Chen X., Kook Y., Ma R., Hu G., Buch S. (2018). Exosomal MiR-9 Released from HIV Tat Stimulated Astrocytes Mediates Microglial Migration. J. Neuroimmune Pharmacol..

[47] Castellani R.J., Lee H.G., Zhu X., Nunomura A., Perry G., Smith M.A. (2006). Neuropathology of Alzheimer Disease: Pathognomonic but Not Pathogenic. Acta Neuropathol..

[48] Kishore U. (2013). Neurodegenerative Diseases.

[49] Carter S.F., Schöll M., Almkvist O., Wall A., Engler H., Långström B., Nordberg A. (2012). Evidence for Astrocytosis in Prodromal Alzheimer Disease Provided by 11C-Deuterium-L-Deprenyl: A Multitracer PET Paradigm Combining 11C-Pittsburgh Compound B and 18F-FDG. J. Nucl. Med..

[50] Wilson H., Dervenoulas G., Pagano G., Tyacke R.J., Polychronis S., Myers J., Gunn R.N., Rabiner E.A., Nutt D. (2019). Imidazoline 2 Binding Sites Reflecting Astroglia Pathology in Parkinson’s Disease: An in Vivo 11C-BU99008 PET Study. Brain.

[51] Poutiainen P., Jaronen M., Quintana F.J., Brownell A., Harvey K. (2016). Precision Medicine in Multiple Sclerosis: Future of PET Imaging of Inflammation and Reactive Astrocytes. Front. Mol. Neurosci..

[52] Linnerbauer M., Wheeler M.A., Quintana F.J. (2020). Astrocyte Crosstalk in CNS Inflammation. Neuron.

[53] Sofroniew M.V. (2020). Astrocyte Reactivity: Subtypes, States, and Functions in CNS Innate Immunity. Trends Immunol..

[54] Santello M., Toni N., Volterra A. (2019). Astrocyte Function from Information Processing to Cognition and Cognitive Impairment. Nat. Neurosci..

[55] Bylicky M.A., Mueller G.P., Day R.M. (2018). Mechanisms of Endogenous Neuroprotective Effects of Astrocytes in Brain Injury. Oxidative Med. Cell. Longev..

[56] Xie K., Qin Q., Long Z., Yang Y., Peng C., Xi C., Li L., Wu Z., Daria V., Zhao Y. (2021). High-Throughput Metabolomics for Discovering Potential Biomarkers and Identifying Metabolic Mechanisms in Aging and Alzheimer’s Disease. Front. Cell Dev. Biol..

[57] Patil S., Chan C. (2005). Palmitic and Stearic Fatty Acids Induce Alzheimer-like Hyperphosphorylation of Tau in Primary Rat Cortical Neurons. Neurosci. Lett..

[58] Ortiz-Rodriguez A., Acaz-Fonseca E., Boya P., Arevalo M.A., Garcia-Segura L.M. (2018). Lipotoxic Effects of Palmitic Acid on Astrocytes Are Associated with Autophagy Impairment. Mol. Neurobiol..

[59] González-Giraldo Y., Garcia-Segura L.M., Echeverria V., Barreto G.E. (2017). Tibolone Preserves Mitochondrial Functionality and Cell Morphology in Astrocytic Cells Treated with Palmitic Acid. Mol. Neurobiol..

[60] González-Giraldo Y., Forero D.A., Echeverria V., Garcia-Segura L.M., Barreto G.E. (2019). Tibolone Attenuates Inflammatory Response by Palmitic Acid and Preserves Mitochondrial Membrane Potential in Astrocytic Cells through Estrogen Receptor Beta. Mol. Cell. Endocrinol..

[61] Liu L., Martin R., Kohler G., Chan C. (2013). Palmitate Induces Transcriptional Regulation of BACE1 and Presenilin by STAT3 in Neurons Mediated by Astrocytes. Exp. Neurol..

[62] Liu L., Martin R., Chan C. (2013). Palmitate-Activated Astrocytes via Serine Palmitoyltransferase Increase BACE1 in Primary Neurons by Sphingomyelinases. Neurobiol. Aging.

[63] Benzinger T.L.S., Blazey T., Jack C.R., Koeppe R.A., Su Y., Xiong C., Raichle M.E., Snyder A.Z., Ances B.M., Bateman R.J. (2013). Regional Variability of Imaging Biomarkers in Autosomal Dominant Alzheimer’s Disease. Proc. Natl. Acad. Sci. USA.

[64] Winocur G., Greenwood C.E. (2005). Studies of the Effects of High Fat Diets on Cognitive Function in a Rat Model. Neurobiol. Aging.

[65] Ho L., Qin W., Pompl P.N., Xiang Z., Wang J., Zhao Z., Peng Y., Cambareri G., Rocher A., Mobbs C.V. (2004). Diet-Induced Insulin Resistance Promotes Amyloidosis in a Transgenic Mouse Model of Alzheimer’s Disease. FASEB J..

[66] Goldman S.E., Goez D., Last D., Naor S., Liraz Zaltsman S., Sharvit-Ginon I., Atrakchi-Baranes D., Shemesh C., Twitto-Greenberg R., Tsach S. (2018). High-Fat Diet Protects the Blood–Brain Barrier in an Alzheimer’s Disease Mouse Model. Aging Cell.

[67] Ghaddar B., Diotel N. (2022). Zebrafish: A New Promise to Study the Impact of Metabolic Disorders on the Brain. Int. J. Mol. Sci..

[68] Booth H.D.E., Hirst W.D., Wade-Martins R. (2017). The Role of Astrocyte Dysfunction in Parkinson’s Disease Pathogenesis. Trends Neurosci..

[69] Deng H., Wang P., Jankovic J. (2018). The Genetics of Parkinson Disease. Ageing Res. Rev..

[70] Chamoli M., Chinta S.J., Andersen J.K. (2018). An Inducible MAO-B Mouse Model of Parkinson’s Disease: A Tool towards Better Understanding Basic Disease Mechanisms and Developing Novel Therapeutics. J. Neural Transm..

[71] Price B.R., Norris C.M., Sompol P., Wilcock D.M. (2018). An Emerging Role of Astrocytes in Vascular Contributions to Cognitive Impairment and Dementia. J. Neurochem..

[72] Arba F., Mair G., Phillips S., Sandercock P., Wardlaw J.M. (2020). Improving Clinical Detection of Acute Lacunar Stroke: Analysis From the IST-3. Stroke.

[73] Qu P., Cheng K., Gao Q., Li Y., Wang M. (2022). Application Value of Serum Hcy, TLR4, and CRP in the Diagnosis of Cerebral Small Vessel Disease. Evidence-based Complement. Altern. Med..

[74] Ponath G., Park C., Pitt D. (2018). The Role of Astrocytes in Multiple Sclerosis. Front. Immunol..

[75] Mayo L., Trauger S.A., Blain M., Nadeau M., Patel B., Alvarez J.I., Mascanfroni I.D., Yeste A., Kivisäkk P., Kallas K. (2014). Regulation of Astrocyte Activation by Glycolipids Drives Chronic CNS Inflammation. Nat. Med..

[76] Sochocka M., Diniz B.S., Leszek J. (2017). Inflammatory Response in the CNS: Friend or Foe?. Mol. Neurobiol..

[77] Ke L., Niu F., Hu G., Yang L., Dallon B., Villareal D., Buch S. (2020). Morphine-Mediated Release OfmiR-138 in Astrocyte-Derived Extracellular Vesicles Promotes Microglial Activation. J Extracell. Vesicles.

[78] Pozo P.H.E., del Espinosa P.S., Donadi E.A., Martinez E.Z., Salazar-Uribe J.C., Guerrero M.A., Moriguti J.C., Colcha M.C., Garcia S.E., Naranjo R. (2018). Cognitive Decline in Adults Aged 65 and Older in Cumbayá, Quito, Ecuador: Prevalence and Risk Factors. Cureus.

[79] Muscat S.M., Deems N.P., D’Angelo H., Kitt M.M., Grace P.M., Andersen N.D., Silverman S.N., Rice K.C., Watkins L.R., Maier S.F. (2021). Postoperative Cognitive Dysfunction Is Made Persistent with Morphine Treatment in Aged Rats. Neurobiol. Aging.

[80] Balaban D., Miyawaki E.K., Bhattacharyya S., Torre M. (2021). The Phenomenon of Clasmatodendrosis. Heliyon.

[81] Fernandes L.M.P., Lopes K.S., Santana L.N.S., Fontes-Júnior E.A., Ribeiro C.H.M.A., Silva M.C.F., de Oliveira Paraense R.S., Crespo-López M.E., Gomes A.R.Q., Lima R.R. (2018). Repeated Cycles of Binge-like Ethanol Intake in Adolescent Female Rats Induce Motor Function Impairment and Oxidative Damage in Motor Cortex and Liver, but Not in Blood. Oxidative Med. Cell. Longev..

[82] Chaudhuri A.D., Dastgheyb R.M., Yoo S.-W., Trout A., Talbot C.C., Hao H., Witwer K.W., Haughey N.J. (2018). TNF Alpha and IL-1 Beta Modify the MiRNA Cargo of Astrocyte Shed Extracellular Vesicles to Regulate Neurotrophic Signaling in Neurons. Cell Death Dis..

[83] Collado-Pérez R., García-Piqueres J., Jiménez-Hernaiz M., Argente J., Belsham D.D., Frago L.M., Chowen J.A. (2021). Fatty Acids Modify the MicroRNA Content of Exosomes Released by Hypothalamic Astrocytes and the Response of POMC Neurons to These Exosomes. J. Endocr. Soc..

[84] Xu L., Cao H., Xie Y., Zhang Y., Du M., Xu X., Ye R., Liu X. (2019). Exosome-Shuttled MiR-92b-3p from Ischemic Preconditioned Astrocytes Protects Neurons against Oxygen and Glucose Deprivation. Brain Res..

[85] Ibáñez F., Montesinos J., Ureña-peralta J.R., Guerri C. (2019). TLR4 Participates in the Transmission of Ethanol-Induced Neuroinflammation via Astrocyte-Derived Extracellular Vesicles. J. Neuroinflamm..

[86] Long X., Yao X., Jiang Q., Yang Y., He X., Tian W., Zhao K., Zhang H. (2020). Astrocyte-Derived Exosomes Enriched with MiR-873a-5p Inhibit Neuroinflammation via Microglia Phenotype Modulation after Traumatic Brain Injury. J. Neuroinflamm..

[87] Du L., Jiang Y., Sun Y. (2021). Astrocyte-Derived Exosomes Carry MicroRNA-17-5p to Protect Neonatal Rats from Hypoxic-Ischemic Brain Damage via Inhibiting BNIP-2 Expression. Neurotoxicology.

[88] Barbosa M., Gomes C., Vaz A.R., Brites D. (2018). Upregulation of MiR-146a Attenuates ALS Mouse Cortical Astrocytes Reactivity and Decrease MiRNA-Inflammatory Associated Exosomal Cargo. Free Radic. Biol. Med..

[89] Yao Y., Wang W., Jing L., Wang Y., Li M., Hou X., Wang J., Peng T., Teng J., Jia Y. (2017). Let-7f Regulates the Hypoxic Response in Cerebral Ischemia by Targeting NDRG3. Neurochem. Res..

[90] Daneshafrooz N., Joghataei M.T., Mehdizadeh M., Alavi A., Barati M., Panahi B., Teimourian S., Zamani B. (2022). Identification of Let-7f and MiR-338 as Plasma-Based Biomarkers for Sporadic Amyotrophic Lateral Sclerosis Using Meta-Analysis and Empirical Validation. Sci. Rep..

[91] Gámez-Valero A., Campdelacreu J., Vilas D., Ispierto L., Reñé R., Álvarez R., Armengol M.P., Borràs F.E., Beyer K. (2019). Exploratory Study on MicroRNA Profiles from Plasma-Derived Extracellular Vesicles in Alzheimer’s Disease and Dementia with Lewy Bodies. Transl. Neurodegener..

[92] Huang H.-T., Hsien H.H., Wu H.-T., Tsai S.-F., Huang H.-Y., Kuo Y.-M., Chen P.-S., Yang C.-S., Tzen S.-F. (2017). High Fat Diet Induces Mitochondria Stress and Impairs Myelin Structure in Rat Hypothalamus. Glia.

[93] Deng Z., Wei Y., Yao Y., Gao S., Wang X. (2020). Let-7f Promotes the Differentiation of Neural Stem Cells in Rats. Am. J. Transl. Res..

[94] Buonfiglioli A., Efe I.E., Guneykaya D., Ivanov A., Huang Y., Orlowski E., Krüger C., Deisz R.A., Markovic D., Flüh C. (2019). Let-7 MicroRNAs Regulate Microglial Function and Suppress Glioma Growth through Toll-Like Receptor 7. Cell Rep..

[95] Yan S., Han X., Xue H., Zhang P., Guo X., Li T., Guo X., Yuan G., Deng L., Li G. (2015). Let-7f Inhibits Glioma Cell Proliferation, Migration, and Invasion by Targeting Periostin. J. Cell. Biochem..

[96] Li K., Wang Z.-Q., Zhang J.-L., Lv P.-Y. (2020). MicroRNA Let-7f Protects against H_2_O_2_-Induced Oxidative Damage in Neuroblastoma Cells by Targeting AKT-2. Arch. Med. Sci..

[97] Shibahara Y., Miki Y., Onodera Y., Hata S., Chan M.S.M., Yiu C.C.P., Loo T.Y., Nakamura Y., Akahira J.I., Ishida T. (2012). Aromatase Inhibitor Treatment of Breast Cancer Cells Increases the Expression of Let-7f, a MicroRNA Targeting CYP19A1. J. Pathol..

[98] Zhao Q.-C., Xu Z.-W., Peng Q.-M., Zhou J.-H., Li Z.-Y. (2020). Enhancement of MiR-16-5p on Spinal Cord Injury-Induced Neuron Apoptosis and Inflammatory Response through Inactivating ERK1/2 Pathway. J. Neurosurg. Sci..

[99] Sun Y., Xiong Y., Yan C., Chen L., Chen D., Mi B., Liu G. (2019). Downregulation of MicroRNA-16-5p Accelerates Fracture Healing by Promoting Proliferation and Inhibiting Apoptosis of Osteoblasts in Patients with Traumatic Brain Injury. Am. J. Transl. Res..

[100] Joilin G., Gray E., Thompson A.G., Bobeva Y., Talbot K., Weishaupt J., Ludolph A., Malaspina A., Leigh P.N., Newbury S.F. (2020). Identification of a Potential Non-Coding RNA Biomarker Signature for Amyotrophic Lateral Sclerosis. Brain Commun..

[101] Wang N., He L., Yang Y., Li S., Chen Y., Tian Z., Ji Y., Wang Y., Pang M., Wang Y. (2020). Integrated Analysis of Competing Endogenous RNA (CeRNA) Networks in Subacute Stage of Spinal Cord Injury. Gene.

[102] Tian F., Yang J., Xia R. (2022). Exosomes Secreted from CircZFHX3-Modified Mesenchymal Stem Cells Repaired Spinal Cord Injury Through Mir-16-5p/IGF-1 in Mice. Neurochem. Res..

[103] You S., He X., Wang M., Mao L., Zhang L. (2020). Tanshinone IIA Suppresses Glioma Cell Proliferation, Migration and Invasion Both In Vitro and In Vivo Partially through MiR-16-5p/Talin-1 (TLN1) Axis. Cancer Manag. Res..

[104] Abyadeh M., Tofigh N., Hosseinian S., Hasan M., Amirkhani A., Fitzhenry M.J., Gupta V., Chitranshi N., Salekdeh G.H., Haynes P.A. (2022). Key Genes and Biochemical Networks in Various Brain Regions Affected in Alzheimer’s Disease. Cells.

[105] Chen D., Dixon B.J., Doycheva D.M., Li B., Zhang Y., Hu Q., He Y., Guo Z., Nowrangi D., Flores J. (2018). IRE1α Inhibition Decreased TXNIP/NLRP3 Inflammasome Activation through MiR-17-5p after Neonatal Hypoxic-Ischemic Brain Injury in Rats. J. Neuroinflamm..

[106] Hong P., Jiang M., Li H. (2014). Functional Requirement of Dicer1 and MiR-17-5p in Reactive Astrocyte Proliferation after Spinal Cord Injury in the Mouse. Glia.

[107] Sajad M., Ahmed M.M., Thakur S.C. (2022). An Integrated Bioinformatics Strategy to Elucidate the Function of Hub Genes Linked to Alzheimer’s Disease. Gene Rep..

[108] Estfanous S., Daily K.P., Eltobgy M., Deems N.P., Anne M.N.K., Krause K., Badr A., Hamilton K., Carafice C., Hegazi A. (2021). Elevated Expression of MiR-17 in Microglia of Alzheimer’s Disease Patients Abrogates Autophagy-Mediated Amyloid-β Degradation. Front. Immunol..

[109] Hébert S.S., Horré K., Nicolaï L., Bergmans B., Papadopoulou A.S., Delacourte A., De Strooper B. (2009). MicroRNA Regulation of Alzheimer’s Amyloid Precursor Protein Expression. Neurobiol. Dis..

[110] Chen B., Yang Y., Wu J., Song J., Lu J. (2021). MicroRNA-17-5p Downregulation Inhibits Autophagy and Myocardial Remodelling after Myocardial Infarction by Targeting STAT3. Autoimmunity.

[111] Wang M., Zhao M., Guo Q., Lou J., Wang L. (2021). Non-Small Cell Lung Cancer Cell–Derived Exosomal MiR-17-5p Promotes Osteoclast Differentiation by Targeting PTEN. Exp. Cell Res..

[112] Qin X., Zhu B., Jiang T., Tan J., Wu Z., Yuan Z., Zheng L., Zhao J. (2019). MiR-17-5p Regulates Heterotopic Ossification by Targeting ANKH in Ankylosing Spondylitis. Mol. Ther. Nucleic Acids.

[113] Xu J., Zhang X., Song X., Tang Y. (2022). Expression of MiR-17-5p in Gastrointestinal Stromal Tumor Tissues and Its Effect on Proliferation and Apoptosis of GIST882 Cells. Chin. J. Cancer Biother..

[114] Deng X.H., Zhong Y., Gu L.Z., Shen W., Guo J. (2013). MiR-21 Involve in ERK-Mediated Upregulation of MMP9 in the Rat Hippocampus Following Cerebral Ischemia. Brain Res. Bull..

[115] Zhou X., Ren Y., Moore L., Mei M., You Y., Xu P., Wang B., Wang G., Jia Z., Pu P. (2010). Downregulation of MiR-21 Inhibits EGFR Pathway and Suppresses the Growth of Human Glioblastoma Cells Independent of PTEN Status. Lab. Investig..

[116] Buller B., Liu X., Wang X., Zhang R.L., Zhang L., Hozeska-Solgot A., Chopp M., Zhang Z.G. (2010). MicroRNA-21 Protects Neurons from Ischemic Death. FEBS J..

[117] Su Y., Chen Z., Du H., Liu R., Wang W., Li H., Ning B. (2019). Silencing MiR-21 Induces Polarization of Astrocytes to the A2 Phenotype and Improves the Formation of Synapses by Targeting Glypican 6 via the Signal Transducer and Activator of Transcription-3 Pathway after Acute Ischemic Spinal Cord Injury. FASEB J..

[118] Zhang L., Dong L.Y., Li Y.J., Hong Z., Wei W.S. (2012). MiR-21 Represses FasL in Microglia and Protects against Microglia-Mediated Neuronal Cell Death Following Hypoxia/Ischemia. Glia.

[119] Feng M.G., Liu C.F., Chen L., Feng W.B., Liu M., Hai H., Lu J.M. (2018). MiR-21 Attenuates Apoptosis-Triggered by Amyloid-β via Modulating PDCD4/PI3K/AKT/GSK-3β Pathway in SH-SY5Y Cells. Biomed. Pharmacother..

[120] Zhao Y., Xu Y., Luo F., Xu W., Wang B., Pang Y., Zhou J., Wang X., Liu Q. (2013). Angiogenesis, Mediated by MiR-21, Is Involved Arsenite-Induced Carcinogenesis. Toxicol. Lett..

[121] Liguori M., Nuzziello N., Introna A., Consiglio A., Licciulli F., D’Errico E., Scarafino A., Distaso E., Simone I.L. (2018). Dysregulation of MicroRNAs and Target Genes Networks in Peripheral Blood of Patients with Sporadic Amyotrophic Lateral Sclerosis. Front. Mol. Neurosci..

[122] Janik P., Fitzgerald J.C., Rai S.N., Huo J., Chen M., Peng L., Gong P., Zheng X., Sun T., Zhang X. (2022). Baicalein Induces Mitochondrial Autophagy to Prevent Parkinson’s Disease in Rats via MiR-30b and the SIRT1/AMPK/MTOR Pathway. Front. Neurol..

[123] Mazzeo A., Lopatina T., Gai C., Trento M., Porta M., Beltramo E. (2019). Functional Analysis of MiR-21-3p, MiR-30b-5p and MiR-150-5p Shuttled by Extracellular Vesicles from Diabetic Subjects Reveals Their Association with Diabetic Retinopathy. Exp. Eye Res..

[124] Guo H., Pu M., Tai Y., Chen Y., Lu H., Qiao J., Wang G., Chen J., Qi X., Huang R. (2020). Nuclear MiR-30b-5p Suppresses TFEB-Mediated Lysosomal Biogenesis and Autophagy. Cell Death Differ..

[125] Shin D., Howng S.Y.B., Ptáček L.J., Fu Y.H. (2012). MiR-32 and Its Target SLC45A3 Regulate the Lipid Metabolism of Oligodendrocytes and Myelin. Neuroscience.

[126] Zhang Y., Wang J., An W., Chen C., Wang W., Zhu C., Chen F., Chen H., Zheng W., Gong J. (2019). MiR-32 Inhibits Proliferation and Metastasis by Targeting EZH2 in Glioma. Technol. Cancer Res. Treat..

[127] Jin Y., Cheng H., Cao J., Shen W. (2019). MicroRNA 32 Promotes Cell Proliferation, Migration, and Suppresses Apoptosis in Colon Cancer Cells by Targeting OTU Domain Containing 3. J. Cell. Biochem..

[128] Scaravilli M., Koivukoski S., Gillen A., Bouazza A., Ruusuvuori P., Visakorpi T., Latonen L. (2022). MiR-32 Promotes MYC-Driven Prostate Cancer. Oncogenesis.

[129] Chen Y.C., Hsu P.Y., Su M.C., Chen T.W., Hsiao C.C., Chin C.H., Liou C.W., Wang P.W., Wang T.Y., Lin Y.Y. (2021). Microrna Sequencing Analysis in Obstructive Sleep Apnea and Depression: Anti-Oxidant and Maoa-Inhibiting Effects of Mir-15b-5p and Mir-92b-3p through Targeting Ptgs1-Nf-Κb-Sp1 Signaling. Antioxidants.

[130] Chen Z., Li Z., Jiang C., Jiang X., Zhang J. (2019). MiR-92b-3p Promotes Neurite Growth and Functional Recovery via the PTEN/AKT Pathway in Acute Spinal Cord Injury. J. Cell. Physiol..

[131] Hao X., Ma C., Chen S., Dang J., Cheng X., Zhu D. (2018). Reverse the down Regulation of MiR-92b-3p by Hypoxia Can Suppress the Proliferation of Pulmonary Artery Smooth Muscle Cells by Targeting USP28. Biochem. Biophys. Res. Commun..

[132] Paul S., Vázquez L.A.B., Uribe S.P., Reyes-Pérez P.R., Sharma A. (2020). Current Status of MicroRNA-Based Therapeutic Approaches in Neurodegenerative Disorders. Cells.

[133] Magri F., Vanoli F., Corti S. (2018). MiRNA in Spinal Muscular Atrophy Pathogenesis and Therapy. J. Cell. Mol. Med..

[134] Kong N., Lu X., Li B. (2014). Downregulation of MicroRNA-100 Protects Apoptosis and Promotes Neuronal Growth in Retinal Ganglion Cells. BMC Mol. Biol..

[135] Wallach T., Mossmann Z.J., Szczepek M., Wetzel M., Machado R., Raden M., Miladi M., Kleinau G., Krüger C., Dembny P. (2021). MicroRNA-100-5p and MicroRNA-298-5p Released from Apoptotic Cortical Neurons Are Endogenous Toll-like Receptor 7/8 Ligands That Contribute to Neurodegeneration. Mol. Neurodegener..

[136] Li X.H., Fu N.S., Xing Z.M. (2019). MiR-100 Suppresses Inflammatory Activation of Microglia and Neuronal Apoptosis Following Spinal Cord Injury via TLR4/NF-ΚB Pathway. Eur. Rev. Med. Pharmacol. Sci..

[137] Wang A.P., Li X.H., Gong S.X., Li W.Q., Hu C.P., Zhang Z., Li Y.J. (2015). MIR-100 Suppresses MTOR Signaling in Hypoxia-Induced Pulmonary Hypertension in Rats. Eur. J. Pharmacol..

[138] Cha D.J., Franklin J.L., Dou Y., Liu Q., Higginbotham J.N., Beckler M.D., Weaver A.M., Vickers K., Prasad N., Levy S. (2015). KRAS-Dependent Sorting of MiRNA to Exosomes. eLife.

[139] Sun C., Liu J., Duan F., Cong L., Qi X. (2022). The Role of the MicroRNA Regulatory Network in Alzheimer’s Disease: A Bioinformatics Analysis. Arch. Med. Sci..

[140] Kit O.I., Pushkin A.A., Alliluyev I.A., Timoshkina N.N., Gvaldin D.Y., Rostorguev E.E., Kuznetsova N.S. (2022). Differential Expression of MicroRNAs Targeting Genes Associated with the Development of High-Grade Gliomas. Egypt. J. Med. Hum. Genet..

[141] Herrero-Aguayo V., Sáez-Martínez P., Jiménez-Vacas J.M., Moreno-Montilla M.T., Montero-Hidalgo A.J., Pérez-Gómez J.M., López-Canovas J.L., Porcel-Pastrana F., Carrasco-Valiente J., Anglada F.J. (2022). Dysregulation of the MiRNome Unveils a Crosstalk between Obesity and Prostate Cancer: MiR-107 Asa Personalized Diagnostic and Therapeutic Tool. Mol. Ther. Nucleic Acids.

[142] Hu G., Liao K., Yang L., Pendyala G., Kook Y., Fox H.S., Buch S. (2017). Tat-Mediated Induction of MiRs-34a &-138 Promotes Astrocytic Activation via Downregulation of SIRT1: Implications for Aging in HAND. J. Neuroimmune Pharmacol..

[143] Madsen P.M., Motti D., Karmally S., Szymkowski D.E., Lambertsen K.L., Bethea J.R., Brambilla R. (2016). Oligodendroglial TNFR2 Mediates Membrane TNF-Dependent Repair in Experimental Autoimmune Encephalomyelitis by Promoting Oligodendrocyte Differentiation and Remyelination. J. Neurosci..

[144] Liu Y., Liu H., Li Y., Mao R., Yang H., Zhang Y., Zhang Y., Guo P., Zhan D., Zhang T. (2020). Circular RNA SAMD4A Controls Adipogenesis in Obesity through the MiR-138-5p/EZH2 Axis. Theranostics.

[145] Devier D.J., Lovera J.F., Lukiw W.J. (2015). Increase in NF-ΚB-Sensitive MiRNA-146a and MiRNA-155 in Multiple Sclerosis (MS) and pro-Inflammatory Neurodegeneration. Front. Mol. Neurosci..

[146] Liu X.S., Chopp M., Pan W.L., Wang X.L., Fan B.Y., Zhang Y., Kassis H., Zhang R.L., Zhang X.M., Zhang Z.G. (2017). MicroRNA-146a Promotes Oligodendrogenesis in Stroke. Mol. Neurobiol..

[147] Gomes C., Cunha C., Nascimento F., Ribeiro J.A., Vaz A.R., Brites D. (2019). Cortical Neurotoxic Astrocytes with Early ALS Pathology and MiR-146a Deficit Replicate Gliosis Markers of Symptomatic SOD1G93A Mouse Model. Mol. Neurobiol..

[148] Huang Z., Liu X., Wu X., Chen M., Yu W. (2022). MiR-146a Alleviates Lung Injury Caused by RSV Infection in Young Rats by Targeting TRAF-6 and Regulating JNK/ERKMAPK Signaling Pathways. Sci. Rep..

[149] Lopez-Ramirez M.A., Wu D., Pryce G., Simpson J.E., Reijerkerk A., King-Robson J., Kay O., De Vries H.E., Hirst M.C., Sharrack B. (2014). MicroRNA-155 Negatively Affects Blood-Brain Barrier Function during Neuroinflammation. FASEB J..

[150] Venkatesha S.H., Dudics S., Song Y., Mahurkar A., Moudgil K.D. (2018). The miRNA Expression Profile of Experimental Autoimmune Encephalomyelitis Reveals Novel Potential Disease Biomarkers. Int. J. Mol. Sci..

[151] Li X., Wang S., Mu W., Barry J., Han A., Carpenter R.L., Jiang B.-H., Peiper S.C., Mahoney M.G., Aplin A.E. (2021). Reactive Oxygen Species Reprogram Macrophages to Suppress Antitumor Immune Response through the Exosomal MiR-155-5p/PD-L1 Pathway. J. Exp. Clin. Cancer Res..

[152] Fei M., Li Z., Cao Y., Jiang C., Lin H., Chen Z. (2021). MicroRNA-182 Improves Spinal Cord Injury in Mice by Modulating Apoptosis and the Inflammatory Response via IKKβ/NF-ΚB. Lab. Investig..

[153] Zhang T., Tian C., Wu J., Zhang Y., Wang J., Kong Q., Mu L., Sun B., Ai T., Wang Y. (2020). MicroRNA-182 Exacerbates Blood-Brain Barrier (BBB) Disruption by Downregulating the MTOR/FOXO1 Pathway in Cerebral Ischemia. FASEB J..

[154] Wang W.M., Lu G., Su X.W., Lyu H., Poon W.S. (2017). MicroRNA-182 Regulates Neurite Outgrowth Involving the PTEN/AKT Pathway. Front. Cell. Neurosci..

[155] Li Z., Zhang L., Liu Z., Huang T., Wang Y., Ma Y., Fang X., He Y., Zhou Y., Huo L. (2020). Research Paper MiRNA-182 Regulated MTSS1 Inhibits Proliferation and Invasion in Glioma Cells. J. Cancer.

[156] Zhou F., Fu W.D., Chen L. (2019). MiRNA-182 Regulates the Cardiomyocyte Apoptosis in Heart Failure. Eur. Rev. Med. Pharmacol. Sci..

[157] Li Y., Luo Y., Li B., Niu L., Liu J., Duan X. (2020). MiRNA-182/Deptor/MTOR Axis Regulates Autophagy to Reduce Intestinal Ischaemia/Reperfusion Injury. J. Cell. Mol. Med..

[158] Nayak B., Khan N., Garg H., Rustagi Y., Singh P., Seth A., Dinda A.K., Kaushal S. (2020). Role of MiRNA-182 and MiRNA-187 as Potential Biomarkers in Prostate Cancer and Its Correlation with the Staging of Prostate Cancer. Int. Braz. J. Urol..

[159] Fu J., Peng L., Tao T., Chen Y., Li Z., Li J. (2019). Regulatory Roles of the MiR-200 Family in Neurodegenerative Diseases. Biomed. Pharmacother..

[160] Higaki S., Muramatsu M., Matsuda A., Matsumoto K., Satoh J.-I., Michikawa M., Niida S. (2018). Defensive Effect of MicroRNA-200b/c against Amyloid-Beta Peptide-Induced Toxicity in Alzheimer’s Disease Models. PLoS ONE.

[161] Yu J., Qin M., Li J., Cui S. (2021). LncRNA SNHG4 Sponges MiR-200b to Inhibit Cell Apoptosis in Diabetic Retinopathy. Arch. Physiol. Biochem..

[162] Huang J., Liang X., Wang J., Kong Y., Zhang Z., Ding Z., Song Z., Guo Q., Zou W. (2019). MiR-873a-5p Targets A20 to Facilitate Morphine Tolerance in Mice. Front. Neurosci..

[163] Fan Y., Li J., Yang Q., Gong C., Gao H., Mao Z., Yuan X., Zhu S., Xue Z. (2019). Dysregulated Long Non-Coding RNAs in Parkinson’s Disease Contribute to the Apoptosis of Human Neuroblastoma Cells. Front. Neurosci..

[164] Lin Y.H., Guo L., Yan F., Dou Z.Q., Yu Q., Chen G. (2020). Long Non-Coding RNA HOTAIRM1 Promotes Proliferation and Inhibits Apoptosis of Glioma Cells by Regulating the MiR-873-5p/ZEB2 Axis. Chin. Med. J..

[165] Pitkänen A., Paananen T., Kyyriäinen J., Das Gupta S., Heiskanen M., Vuokila N., Bañuelos-Cabrera I., Lapinlampi N., Kajevu N., Andrade P. (2021). Biomarkers for Posttraumatic Epilepsy. Epilepsy Behav..

[166] Califf R.M. (2018). Minireview Biomarker Definitions and Their Applications. Exp. Biol. Med..

[167] Majkić-Singh N. (2011). What Is a Biomarker? From Its Discovery to Clinical Application. J. Med. Biochem..

[168] Younas N., Flores L.C.F., Hopfner F., Höglinger G.U., Zerr I. (2022). A New Paradigm for Diagnosis of Neurodegenerative Diseases: Peripheral Exosomes of Brain Origin. Transl. Neurodegener..

[169] Brownlee W.J. (2019). Misdiagnosis of Multiple Sclerosis. Neurology.

[170] Happich M., Kirson N.Y., Desai U., King S., Birnbaum H.G., Reed C., Belger M., Lenox-Smith A., Price D. (2016). Excess Costs Associated with Possible Misdiagnosis of Alzheimer’s Disease among Patients with Vascular Dementia in a UK CPRD Population. J. Alzheimer’s Dis..

[171] Tang W., Hu Z., Muallem H., Gulley M.L. (2011). Clinical Implementation of RNA Signatures for Pharmacogenomic Decision-Making. Pharmgenom. Pers. Med..

[172] Qian Y., Daza J., Itzel T., Betge J., Zhan T., Marm F., Teufel A. (2021). Prognostic Cancer Gene Expression Signatures: Current Status and Challenges. Cells.

[173] Tan Y.J., Wong B.Y.X., Vaidyanathan R., Sreejith S., Chia S.Y., Kandiah N., Ng A.S.L., Zeng L. (2021). Altered Cerebrospinal Fluid Exosomal MicroRNA Levels in Young-Onset Alzheimer’s Disease and Frontotemporal Dementia. J. Alzheimer’s Dis. Rep..

[174] Li H., Hong G., Lin M., Shi Y., Wang L., Jiang F., Zhang F., Wang Y., Guo Z. (2017). Identification of Molecular Alterations in Leukocytes from Gene Expression Profiles of Peripheral Whole Blood of Alzheimer’s Disease. Sci. Rep..

[175] Gharbi T., Zhang Z., Yang G.Y. (2020). The Function of Astrocyte Mediated Extracellular Vesicles in Central Nervous System Diseases. Front. Cell Dev. Biol..

[176] Chim S.S.C., Shing T.K.F., Hung E.C.W., Leung T.Y., Lau T.K., Chiu R.W.K., Lo Y.M.D. (2008). Detection and Characterization of Placental MicroRNAs in Maternal Plasma. Clin. Chem..

[177] Mitchell P.S., Parkin R.K., Kroh E.M., Fritz B.R., Wyman S.K., Pogosova-Agadjanyan E.L., Peterson A., Noteboom J., O’Briant K.C., Allen A. (2008). Circulating MicroRNAs as Stable Blood-Based Markers for Cancer Detection. Proc. Natl. Acad. Sci. USA.

[178] Vidal M. (2019). Exosomes: Revisiting Their Role as “Garbage Bags". Traffic.

[179] Meldolesi J. (2021). Extracellular Vesicles (Exosomes and Ectosomes) Play Key Roles in the Pathology of Brain Diseases. Mol. Biomed..

[180] Mussbacher M., Pirabe A., Brunnthaler L., Schrottmaier W.C., Assinger A. (2021). Horizontal MicroRNA Transfer by Platelets—Evidence and Implications. Front. Physiol..

[181] Mao S., Sun Q., Xiao H., Zhang C., Li L. (2015). Secreted MiR-34a in Astrocytic Shedding Vesicles Enhanced the Vulnerability of Dopaminergic Neurons to Neurotoxins by Targeting Bcl-2. Protein Cell.

[182] Battistelli M., Falcieri E. (2020). Apoptotic Bodies: Particular Extracellular Vesicles Involved in Intercellular Communication. Biology.

[183] Valle-Tamayo N., Pérez-González R., Chiva-Blanch G., Belbin O., Serrano-Requena S., Sirisi S., González A.C., Giró O., Sánchez-Aced É., Dols-Icardo O. (2022). Enrichment of Astrocyte-Derived Extracellular Vesicles from Human Plasma. J. Vis. Exp..

[184] Dickens A.M., Tovar-Y-Romo L.B., Yoo S.-W.W., Trout A.L., Bae M., Kanmogne M., Megra B., Williams D.W., Witwer K.W., Gacias M. (2017). Astrocyte-Shed Extracellular Vesicles Regulate the Peripheral Leukocyte Response to Inflammatory Brain Lesions. Sci. Signal..

[185] García-Romero N., Carrión-Navarro J., Esteban-Rubio S., Lázaro-Ibáñez E., Peris-Celda M., Alonso M.M., Guzmán-De-Villoria J., Fernández-Carballal C., de Mendivil A.O., García-Duque S. (2017). DNA Sequences within Glioma-Derived Extracellular Vesicles Can Cross the Intact Blood-Brain Barrier and Be Detected in Peripheral Blood of Patients. Oncotarget.

[186] Chung N.S., Wasan K.M. (2004). Potential Role of the Low-Density Lipoprotein Receptor Family as Mediators of Cellular Drug Uptake. Adv. Drug Deliv. Rev..

[187] Wei Z., Batagov A.O., Schinelli S., Wang J., Wang Y., El Fatimy R., Rabinovsky R., Balaj L., Chen C.C., Hochberg F. (2017). Coding and Noncoding Landscape of Extracellular RNA Released by Human Glioma Stem Cells. Nat. Commun..

[188] Bayraktar R., Van Roosbroeck K., Calin G.A. (2017). Cell-to-cell Communication: MicroRNAs as Hormones. Mol. Oncol..

[189] Dehouck B., Fenart L., Dehouck M.P., Pierce A., Torpier G., Cecchelli R. (1997). A New Function for the LDL Receptor: Transcytosis of LDL across the Blood–Brain Barrier. J. Cell Biol..

[190] Rhea E.M., Banks W.A. (2021). Interactions of Lipids, Lipoproteins, and Apolipoproteins with the Blood-Brain Barrier. Pharm. Res..

[191] You Y., Muraoka S., Jedrychowski M.P., Hu J., McQuade A.K., Young-Pearse T., Aslebagh R., Shaffer S.A., Gygi S.P., Blurton-Jones M. (2022). Human Neural Cell Type-Specific Extracellular Vesicle Proteome Defines Disease-Related Molecules Associated with Activated Astrocytes in Alzheimer’s Disease Brain. J. Extracell. Vesicles.

[192] Gosselin R.-D., Meylan P., Decosterd I. (2013). Extracellular Microvesicles from Astrocytes Contain Functional Glutamate Transporters: Regulation by Protein Kinase C and Cell Activation. Front. Cell. Neurosci..

[193] Théry C., Witwer K.W., Aikawa E., Alcaraz M.J., Anderson J.D., Andriantsitohaina R., Antoniou A., Arab T., Archer F., Atkin-Smith G.K. (2018). Minimal Information for Studies of Extracellular Vesicles 2018 (MISEV2018): A Position Statement of the International Society for Extracellular Vesicles and Update of the MISEV2014 Guidelines. J. Extracell. Vesicles.

[194] Gerstberger S., Hafner M., Tuschl T. (2014). A Census of Human RNA-Binding Proteins. Nat. Rev. Genet..

[195] Galvanin A., Dostert G., Ayadi L., Marchand V., Motorin Y. (2019). Diversity and Heterogeneity of Extracellular RNA in Human Plasma. Biochimie.

[196] Shurtleff M.J., Yao J., Qin Y., Nottingham R.M., Temoche-Diaz M.M., Schekman R., Lambowitz A.M. (2017). Broad Role for YBX1 in Defining the Small Noncoding RNA Composition of Exosomes. Proc. Natl. Acad. Sci. USA.

[197] Zhao Y., Gan Y., Xu G., Hua K., Liu D. (2020). Exosomes from MSCs Overexpressing MicroRNA-223-3p Attenuate Cerebral Ischemia through Inhibiting Microglial M1 Polarization Mediated Inflammation. Life Sci..

[198] Hu G., Yao H., Chaudhuri A.D., Duan M., Yelamanchili S.V., Wen H., Cheney P.D., Fox H.S., Buch S. (2012). Exosome-Mediated Shuttling of MicroRNA-29 Regulates HIV Tat and Morphine-Mediated Neuronal Dysfunction. Cell Death Dis..

[199] You Y., Borgmann K., Edara V.V., Stacy S., Ghorpade A., Ikezu T. (2020). Activated Human Astrocyte-Derived Extracellular Vesicles Modulate Neuronal Uptake, Differentiation and Firing. J. Extracell. Vesicles.

[200] Morioka K., Marmor Y., Sacramento J.A., Lin A., Shao T., Miclau K.R., Clark D.R., Beattie M.S., Marcucio R.S., Miclau T. (2019). Differential Fracture Response to Traumatic Brain Injury Suggests Dominance of Neuroinflammatory Response in Polytrauma. Sci. Rep..

[201] Watson C.N., Belli A., Di Pietro V. (2019). Small Non-Coding RNAs: New Class of Biomarkers and Potential Therapeutic Targets in Neurodegenerative Disease. Front. Genet..

[202] Khan N.Z., Cao T., He J., Ritzel R.M., Li Y., Henry R.J., Colson C., Stoica B.A., Faden A.I., Wu J. (2021). Spinal Cord Injury Alters MicroRNA and CD81+ Exosome Levels in Plasma Extracellular Nanoparticles with Neuroinflammatory Potential. Brain. Behav. Immun..

[203] Gupta S., Rawat S., Arora V., Kottarath S.K., Dinda A.K., Vaishnav P.K., Nayak B., Mohanty S. (2018). An Improvised One-Step Sucrose Cushion Ultracentrifugation Method for Exosome Isolation from Culture Supernatants of Mesenchymal Stem Cells. Stem Cell Res. Ther..

[204] Osti D., del Bene M., Rappa G., Santos M., Matafora V., Richichi C., Faletti S., Beznoussenko G.V., Mironov A., Bachi A. (2019). Clinical Significance of Extracellular Vesicles in Plasma from Glioblastoma Patients. Clin. Cancer Res..

